# FUT8-mediated aberrant N-glycosylation of SEMA7A promotes head and neck squamous cell carcinoma progression

**DOI:** 10.1038/s41368-024-00289-w

**Published:** 2024-03-28

**Authors:** Zhonglong Liu, Xiaoyan Meng, Yuxin Zhang, Jingjing Sun, Xiao Tang, Zhiyuan Zhang, Liu Liu, Yue He

**Affiliations:** 1 Department of Oral Maxillofacial & Head and Neck Oncology, Shanghai Ninth People’s Hospital Affiliated to Shanghai Jiao Tong University School of Medicine, National Clinical Research Center for Oral Disease, Shanghai, China; 2 Department of Oral Surgery, Shanghai Ninth People’s Hospital, Shanghai Jiao Tong University School of Medicine; College of Stomatology, Shanghai Jiao Tong University; National Center for Stomatology; National Clinical Research Center for Oral Diseases, Shanghai, China; 3 Department of Oral Pathology, Shanghai Ninth People’s Hospital Affiliated to Shanghai Jiao Tong University School of Medicine, National Clinical Research Center for Oral Disease Shanghai, Shanghai, China

**Keywords:** Oral cancer, Cancer microenvironment

## Abstract

SEMA7A belongs to the Semaphorin family and is involved in the oncogenesis and tumor progression. Aberrant glycosylation has been intricately linked with immune escape and tumor growth. SEMA7A is a highly glycosylated protein with five glycosylated sites. The underlying mechanisms of SEMA7A glycosylation and its contribution to immunosuppression and tumorigenesis are unclear. Here, we identify overexpression and aberrant N-glycosylation of SEMA7A in head and neck squamous cell carcinoma, and elucidate fucosyltransferase FUT8 catalyzes aberrant core fucosylation in SEMA7A at N-linked oligosaccharides (Asn 105, 157, 258, 330, and 602) via a direct protein‒protein interaction. A glycosylated statue of SEMA7A is necessary for its intra-cellular trafficking from the cytoplasm to the cytomembrane. Cytokine EGF triggers SEMA7A N-glycosylation through increasing the binding affinity of SEMA7A toward FUT8, whereas TGF-β1 promotes abnormal glycosylation of SEMA7A via induction of epithelial–mesenchymal transition. Aberrant N-glycosylation of SEMA7A leads to the differentiation of CD8^+^ T cells along a trajectory toward an exhausted state, thus shaping an immunosuppressive microenvironment and being resistant immunogenic cell death. Deglycosylation of SEMA7A significantly improves the clinical outcome of EGFR-targeted and anti-PD-L1-based immunotherapy. Finally, we also define RBM4, a splice regulator, as a downstream effector of glycosylated SEMA7A and a pivotal mediator of PD-L1 alternative splicing. These findings suggest that targeting FUT8-SEMA7A axis might be a promising strategy for improving antitumor responses in head and neck squamous cell carcinoma patients.

## Introduction

Head and neck squamous cell carcinoma (HNSCC), with 1 000 000 new cases annually, is the sixth most common cancer worldwide.^1^ Despite the implementation of comprehensive and sequential therapy, a high percentage (50%–60%) of advanced HNSCC patients experience relapse or lymph node/distant metastasis. Elucidating the underlying mechanism of HNSCC progression and thus improving the clinical outcome of targeted and immunotherapies, is a herculean task. Glycosylation, an indispensable type of posttranslational modification (PTM), plays a crucial role in regulating cell- and microenvironment-specific processes under physiological and disease conditions. Aberrant glycosylation, orchestrated by altered glycotransferase and glycosidase expression, aberrant glycan structures and lectins, de novo synthesis of terminal sialylated glycans, and fucosylation, has been identified to be involved in extracellular matrix modulation (ECM), cellular interactions, and immune recognition and response. Cumulative evidence shows that aberrant glycosylation accompanies the acquisition of molecular and cellular characteristics necessary for tumor progression and metastasis.^2–7^ For example, regarding immunotherapy, PD-L1 glycosylation suppresses T-cell activity, thus lessening the therapeutic efficacy of PD-1 blockade.^8–10^ Eradication of N-linked glycosylation enhances PD-L1 exposure and abrogates the PD-L1/PD-1 interaction, thus augmenting the efficiency of immune checkpoint therapy.^11–13^ Similar biological phenomena have also been found for B7H3, B7H4, PD-L2, PD-1, EGFR and so on.^10,14–18^ These discoveries indicated inhibition of aberrant glycosylation as a step toward a novel antitumor therapy.

SEMA7A, also named CD108w, belongs to the Semaphorin family; it contains two cell surface receptors composed of integrin β1 and α1 or αV subunits and plexinC1, and is well known for its role in angiogenesis, olfactory nerve outgrowth and the immune response.^19–24^ The association of SEMA7A with oncogenesis and tumor progression has been increasingly clarified. SEMA7A drives breast cancer progression through pleiotropic effects, inhibition of mesenchymal factors, and promotion of macrophage-mediated lymphatic remodeling.^25–28^ In EGFR-mutant lung adenocarcinoma cells, SEMA7A induces EGFR-TKI resistance through ERK activation and apoptosis inhibition.^29^ Few studies have investigated the mechanism of SEMA7A in the initiation and progression of HNSCC. Through RNA-seq of HNSCC samples and validation with a tissue microarray, we identified the overexpression of SEMA7A in tumor samples and the subsequent poorer clinical outcome. In this respect, we speculated that SEMA7A may play a pivotal role in HNSCC progression. Moreover, SEMA7A is found mostly on the plasma membrane and is a glycosylphosphatidylinositol-anchored (GPI-anchored) enzyme with five putative N-linked glycosylation sites. However, biological effects of SEMA7A glycosylation have rarely been elucidated, nor have the molecular mechanisms that regulate the N-glycosylation of SEMA7A and subsequently affect tumor progression.

Here, we explore the complex function of aberrant N-glycosylation of SEMA7A in the oncogenesis of HNSCC. We show that the fucosyltransferase FUT8 catalyzes aberrant core fucosylation in SEMA7A at N-linked oligosaccharides (Asn 105, 157, 258, 330, and 602) via a direct protein‒protein interaction, an action that is necessary for SEMA7A trafficking from the cytoplasm to the cytomembrane and indispensable for HNSCC progression. Furthermore, EGF and TGF-β1 induce SEMA7A N-glycosylation via different mechanisms; specifically, EGF significantly increases the binding activity of SEMA7A-WT toward FUT8, whereas TGF-β1 promotes SEMA7A N-glycosylation through induction of epithelial-mesenchymal transition. Aberrant N-glycosylation of SEMA7A leads to the differentiation of CD8^+^ T cells along a trajectory toward an exhausted state, thus shaping an immunosuppressive microenvironment. Deglycosylation of SEMA7A significantly improves the clinical outcome of EGFR-targeted and anti-PD-L1-based immunotherapy. Finally, we also define RBM4, a splice regulator, as a downstream effector of glycosylated SEMA7A and a pivotal mediator of PD-L1 alternative splicing. The abovementioned results may provide a solid foundation for uncovering the oncogenic role of aberrant glycosylation of SEMA7A in HNSCC, thus further improving therapeutic efficacy.

## Results

### Involvement of SEMA7A overexpression in HNSCC progression

Through RNA-Seq analysis of HNSCC samples (3 tumors and 3 para-carcinoma tissues), we identified 8 pivotal marker genes (PCDH7, CDH2, ITGA3, SEMA7A, TMEM132A, CD276, TMEM2, GPR158) that were overexpressed in tumor specimens (Supplementary Fig. 1a, Supplementary Table 1). Subsequently, high enrichment of these molecules was further verified in 5 HNSCC cell lines, along with the finding that SEMA7A presented marked upregulation in all cell types (Supplementary Fig. 1b, c), indicating its participatory role in tumor progression. However, the underlying mechanism of SEMA7A in HNSCC is poorly defined.

**Fig. 1:**
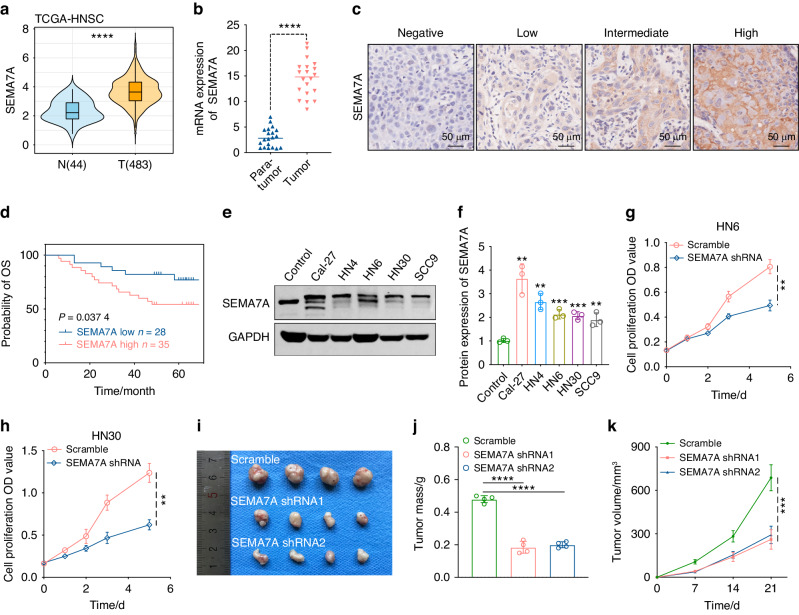
Identification of the promotive role of SEMA7A in HNSCC progression. **a** Distinct transcriptive level of SEMA7A between HNSCC tumors and adjacent normal tissues in HNSCC-TCGA database. **b** Verification of the transcriptive divergence in clinical specimens of HNSCC (20 para-tumors vs. 20 tumors). **c** The representative IHC images with diverse intensities (negative, low, intermediate and high) staining in tissue microarray from HNSCC tumors. **d** Kaplan–Meier plots of the overall survival of HNSCC patients stratified by the IHC score of SEMA7A (*p* value was calculated using the log-rank test). **e**, **f** Expression of SEMA7A protein in five representative HNSCC cell lines by immunoblot (**e**) and semi-quantitative analysis (**f**). **g**, **h** Cell proliferation measurement in HN6 (**g**) and HN30 (**h**) cell lines transfected with scramble and SEMA7A-shRNA, respectively. **i**–**k**. Confirmation of SEMA7A knockdown-mediated tumor growth inhibition in subcutaneous xenograft model (*n* = 4) presented as general review (**i**), tumor weight (**j**) and growth curve (**k**). (Data were shown as mean ± SEM, **P ≤ 0.01, ***P ≤ 0.001, ****P ≤ 0.000 1)

SEMA7A is widely expressed in human tissues and organs, such as the vitreous humor, colonic epithelial cells, and testis (Supplementary Fig. 2a). In addition, SEMA7A is highly expressed across different cancer types (for example, HNSCC), with overexpression in tumor tissues compared to paired para-cancerous samples (Supplementary Fig. 2b). Significant upregulation of SEMA7A in the oral cavity, oropharynx and hypopharynx was also discovered by analysis of the TCGA-HNSCC database (Fig. 1a, Supplementary Fig. 2c). This was further validated by polymerase chain reaction (PCR) analyses in fresh HNSCC tumor tissues and precancerous lesions (20 vs. 20) (Fig. 1b). A high abundance of SEMA7A in tumors indicated a poorer clinical outcome of overall survival (OS) in the TCGA database (Supplementary Fig. 2d). Ultimately, tissue microarray analysis (TMA) with 63 HNSCC samples confirmed the positive correlation of higher expression of SEMA7A in tumor cells (high: positive ratio ≥ 20%; low: positive ratio <20%) with poorer prognosis (Fig. 1c, d).

**Fig. 2:**
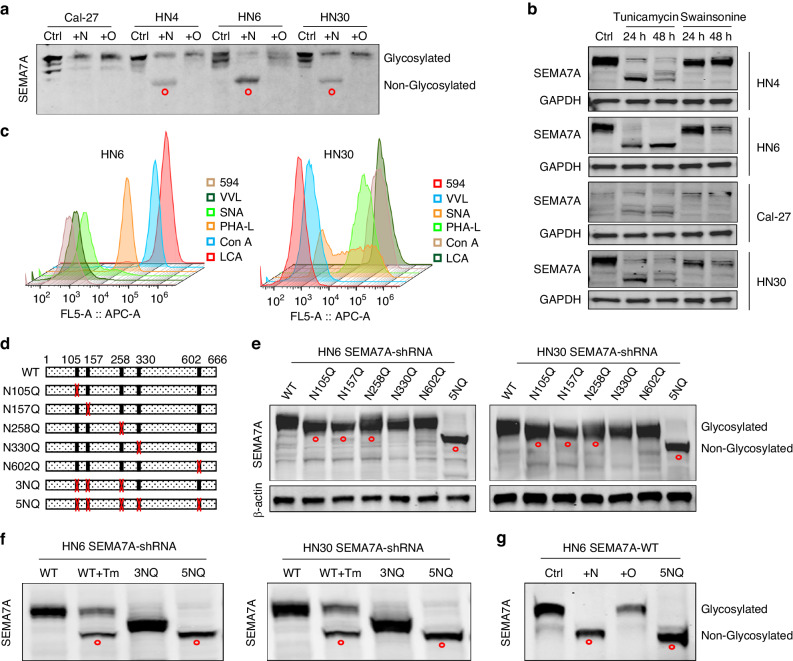
Aberrant N-glycosylation of SEMA7A in HNSCC cell lines. **a** Western blot analysis of SEMA7A (glycosylated and non-glycosylated form) in four HNSCC cell lines pre-treated with or without PNGase F (+N) and O-Glycosidase (+O). **b** Western blot analysis of SEMA7A in HNSCC cell lines pre-incubated with tunicamycin and swainsonine at the designed timepoint of 24 and 48 h. **c** Flow cytometry measuring of different lectins (VVL, SNA, PHA-L, Con A and LCA) on the membrane of HN6 (left) and HN30 (right) cells. **d** Schematic diagram of mutation plasmids construction based on the five glycosylation sites of SEMA7A by substituting of asparagines (N) to glutamine (Q). **e** Verification of the exogenous expression and deglycosylated statues of wild-type SEMA7A or the single-site N-glycosylation SEMA7A mutant in HN6 (left) and HN30 (right) cells transfected with SEMA7A-shRNA. **f** Comparison of the deglycosylated efficiency of tunicamycin with SEMA7A-3NQ and SEMA7A-5NQ mutants in HN6 (left) and HN30 (right) cells. **g** Comparison of the deglycosylated statue in HN6 cells treated with PNGase F (+N), O-Glycosidase (+O) and SEMA7A-5NQ mutation plasmid

To explore the oncogenic potential of SEMA7A in HNSCC, we carried out in vitro and in vivo verification analyses. Through protein and mRNA level measurement, we finally selected the HN6 and HN30 cell lines as the major objects for further investigation (Fig. 1e, f, Supplementary Fig. 2e). *V*ia a knockdown strategy, we established stable clones with SEMA7A knockdown with ideal knockdown efficiency in HN6 and HN30 cell lines by using a lentiviral vector. In vitro cell proliferation experiments demonstrated an obviously lower proliferation rate in SEMA7A knockdown clones (Fig. 1g, h). To further confirm this growth inhibition in vivo, HN6 knockdown cells were injected into the flank region of BALB/c nude mice, and downregulation of SEMA7A was found to significantly suppress the tumorigenesis of HN6 cells in vivo, as evidenced by the tumor mass and growth curve (Fig. 1i–k). Collectively, the current data support the concept that SEMA7A is an oncoprotein in HNSCC.

### Evaluating the glycosylation of SEMA7A in HNSCC

When detecting the protein expression of SEMA7A in different HNSCC cell lines, we found a dramatic band shift toward a higher molecular weight in tumor cells compared with normal epithelia (Fig. 1e), suggesting the presence of posttranslational modifications, especially glycosylation, which is intimately correlated with the molecular weight of SEMA7A. Incubation with peptide-N-glycosidase F (PNGase F) to remove N-glycosylation (deglycosylation) demonstrated band separation to a nonglycosylated form, which was not observed after treatment with O-glycosidase to remove O-glycans (Fig. 2a). Subsequently, incubation of tumor cells with tunicamycin, a well-known inhibitor of N-glycosylation, also showed results similar to those observed after PNGase F treatment; however, this protein band shift was not detected in swainsonine incubated group (Fig. 2b). As mentioned above, this band shift may be ascribed to the aberrant N-glycosylation of SEMA7A in cancer cells. To determine the specific patterns of N-glycosylation, we performed flow cytometry by using anti-lectin antibodies (*Vicia villosa* lectin, VVL; *Sambucus nigra* lectin, SNA; *Phaseolus vulgaris* leucoagglutinin, PHA-L; *Concanavalin* A, Con A; *Lens culinaris* agglutinin, LCA) and revealed that LCA was the dominant form of SEMA7A N-glycosylation in both HN6 and HN30 cells (Fig. 2c).

To delineate the molecular mechanism by which aberrant glycosylation of SEMA7A promotes HNSCC oncogenesis, we conducted glycosylation intervention experiments by constructing mutant plasmids. Through amino acid sequence analysis, five NXT motif sites were found in human SEMA7A (Asn 105, 157, 258, 330, and 602) (Fig. 2d). We then generated the SEMA7A-WT and SEMA7A-5NQ mutants by substituting asparagine (N) with glutamine (Q). The effect of deglycosylation was validated in SEMA7A-shRNA cell models (Fig. 2e). Samples containing the N105Q, N157Q, and N258Q proteins showed a slight band shift that was not found in cells with N258Q and N602Q. To elucidate whether the band shift in 5NQ was attributed to glycosylation of residues 105, 157, and 258, we reconstructed the 3NQ mutant plasmid and found that all mutations in glycosylated sites contributed to deglycosylation (Fig. 2f). Compared with the deglycosylation efficiency of tunicamycin and PNGase F treatment, the 5NQ mutation completely ablated SEMA7A N-glycosylation, as indicated by the identical band shift (Fig. 2g).

### Glycosylation status determined the protein degradation patterns

**Fig. 3:**
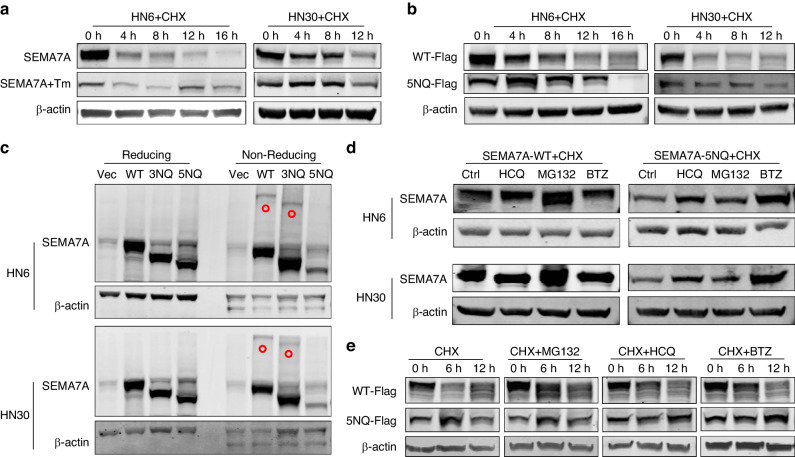
Distinct statues of glycosylation determined the SEMA7A protein degradation manner. **a**, **b** The intensity of endogenous (**a**) and exogenous (**b**) SEMA7A protein in HN6 (left) and HN30 (right) cells pre-treated with CHX (20 μmol/L) at designed time intervals in the presence of 2.5 μg/mL tunicamycin or not. **c** Detection of SEMA7A protein dimer formation in HN6 (up) and HN30 (down) cells transfected with different mutation plasmids at reducing and non-reducing conditions. **d** Western blot analysis of SEMA7A in HN6 (up) and HN30 (down) cells exogenously expressing either wild type (left) or 5NQ mutant (right) in the presence of CHX (20 μmol/L for 12 h) and subsequent HCQ, MG132 and BTZ for 6 h. **e** Protein expression of exogenous Flag-tag in HNSCC cells treated with CHX, and followed by HCQ, MG132, and BTZ at indicated time intervals

Glycosylation has been reported to participate in protein degradation and stability. After treatment with the protein synthesis inhibitor cycloheximide (CHX), endogenous glycosylated SEMA7A exhibited a higher turnover rate than the SEMA7A protein with deglycosylation induced by tunicamycin (Fig. 3a). This difference in degradation rate was further confirmed by evaluation of exogenous SEMA7A-WT and SEMA7A-5NQ in the presence of CHX (Fig. 3b, Supplementary Fig. 3a). These data indicated a more stable status of nonglycosylated SEMA7A, with less susceptibility to degradation. Subsequently, we investigated the underlying mechanism to interpret this biological phenomenon. Under nonreducing conditions, which had no significant influence on that of SEMA7A-WT and SEMA7A-3NQ, SEMA7A-5NQ lost the capability to form protein dimers, implying dysfunction in SEMA7A-5NQ signal transduction (Fig. 3c). Protein degradation is accomplished through three main pathways: (1) the ubiquitin‒proteasome system (UPS); (2) autophagic degradation; and (3) endoplasmic reticulum-associated degradation (ERAD). By incubation with inhibitors (MG132 for the UPS, hydroxychloroquine (HCQ) for autophagy and bortezomib (BTZ) for ERAD), we found completely distinct degradation pathways between nonglycosylated (through ERAD) and glycosylated (via the UPS) SEMA7A, as evidenced by the different protein accumulation patterns observed following treatment with the degradation inhibitors (Fig. 3d). This degradation trend was further proven by immunoblot analysis of exogenous Flag-tagged SEMA7A proteins at different timepoints of 0, 6 and 12 h (Fig. 3e). To further verify the difference in UPS-dependent proteolysis, cells were sequentially transfected with ubiquitin and SEMA7A-WT/5NQ and then incubated with tunicamycin and MG132. The data showed that the UPS system failed to facilitate the degradation of ubiquitinated SEMA7A-5NQ (Supplementary Fig. 3b). Conversely, cells expressing SEMA7A-5NQ demonstrated relatively lower activity of enzymes involved in ERAD, such as OS9 and ERECL1, which indicated that ERAD-dependent degradation was suppressed when SEMA7A was deglycosylated, thus leading to relatively higher protein stability (Supplementary Fig. 3c). We also speculated that the secreted form of SEMA7A is correlated with this difference in degradation. However, no difference was found between SEMA7A-WT- and 5NQ-overexpressing cells by using ELISA (Supplementary Fig. 3d). Thus, the more stable state of nonglycosylated SEMA7A may contribute to the lower activity of ERAD enzymes.

### N-glycosylation of SEMA7A promoted HNSCC progression

**Fig. 4:**
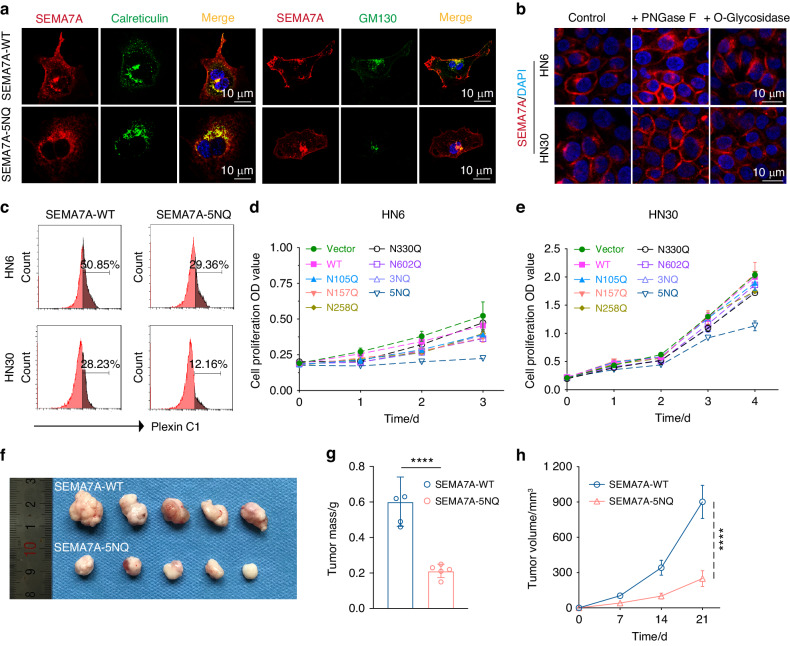
Aberrant glycosylation of SEMA7A facilitated HNSCC progression. **a** Confocal microscopy images of co-distribution of SEMA7A with Endoplasmic Reticulum (calreticulin, left) and Golgi markers (GM130, right) in HNSCC cells transfected with WT (up) and 5NQ (down) plasmids. **b** Immunofluorescence observation of SEMA7A distribution and intensity in HN6 (up) and HN30 (down) cells pre-treated with PNGase F and O-Glycosidase. **c** Flow cytometry analysis of the binding activity between SEMA7A-WT/ Plexin C1 and SEMA7A-5NQ/ Plexin C1 in HN6 (up) and HN30 (down) cells. **d**, **e** Diverse cellular proliferation rates of HN6 (**d**) and HN30 (**e**) cells transfected with various mutation plasmids detecting by CCK-8. **f**–**h** Tumor growth of SEMA7A-WT and 5NQ HN6 cells in BALB/c nude mice, showing as gross view of isolated tumors (**f**), tumor mass (**g**), and growth curve (**h**). Data was shown as the mean ± SD (*n* = 5), *P* value was calculated by Student’s *t* test (****P < 0.000 1)

To clarify whether glycosylation of SEMA7A is indispensable for HNSCC oncogenesis, in vitro and in vivo functional alterations were explored. The subcellular distribution is a pivotal factor in the ability of proteins to execute their precise biological functions. N-glycosylation was necessary for SEMA7A trafficking from the cytoplasm to the cytomembrane, as evidenced by the increased colocalization of SEMA7A-5NQ with endoplasmic reticulum (calreticulin) and Golgi (GM130) markers compared to that of SEMA7A-WT observed by laser confocal microscopy (LCFM), indicating retention of the SEMA7A-5NQ molecule in the cytoplasm (Fig. 4a). The contribution of individual N-glycosylation sites to SEMA7A subcellular localization was also determined by LCFM, with the finding that none of the single-site mutations had an influence on its cytomembrane trafficking (Supplementary Fig. 4a). Removal of N-glycosylation enhanced the detection of SEMA7A, as proven by the significant increase in the fluorescence intensity of SEMA7A in both the HN6 and HN30 cytomembranes compared with that in the control and O-glycan removal treatment groups (Fig. 4b). Plexin C1 is a classical receptor with high affinity for SEMA7A.^30,31^ We then explored whether deglycosylation of SEMA7A affected the formation of the SEMA7A/Plexin C1 complex. SEMA7A-WT/5NQ cells were incubated with recombinant Plexin C1 protein (50 µg/ml), and the affinity difference was assessed by flow cytometry. Unsurprisingly, complete ablation of N-glycosylation significantly attenuated the binding of SEMA7A to Plexin C1 (Fig. 4c, Supplementary Fig. 4b).

Whether impairment of SEMA7A N-glycosylation blocks tumor progression is also a pivotal issue. In vitro proliferation analysis demonstrated that deglycosylation of SEMA7A dramatically decreased cell viability and was negatively correlated with tumor invasion in both HN6 and HN30 cells (Fig. 4d, e, Supplementary Fig. 5). These results were further confirmed in an in vivo mouse model that showed a decreased tumor mass and volume (Fig. 4f–h). Taken together, these data demonstrated an important role of SEMA7A N-glycosylation in tumorigenesis.

**Fig. 5:**
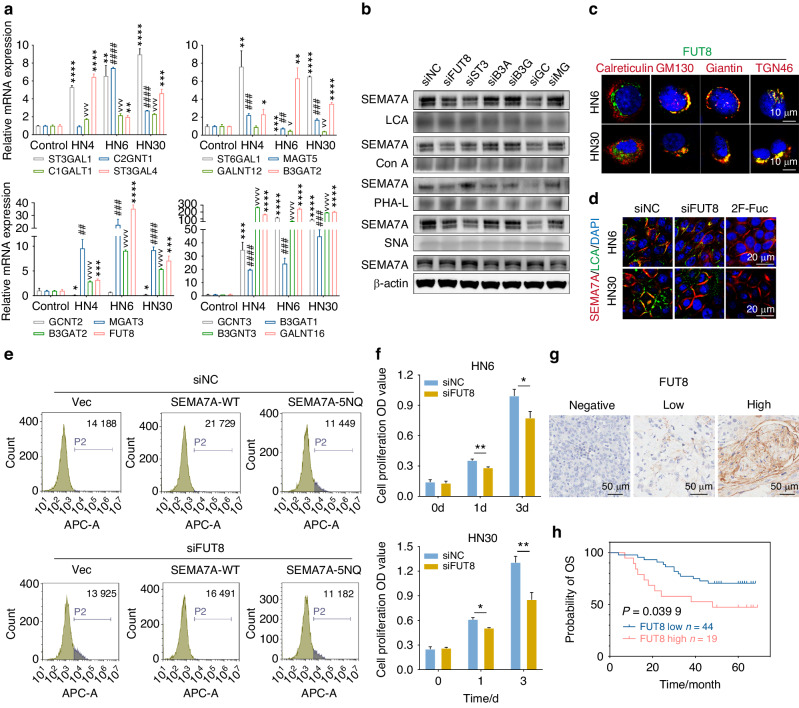
Identification of FUT8 as glycotransferases for SEMA7A aberrant glycosylation. **a** qRT-PCR analysis of 16 N-glycosyltransferases mRNA expression in HNSCC cell lines (HN4, HN6, and HN30). **b** Lectin pull-down analysis (LCA, Con A, PHA-L, SNA) of SEMA7A in HNSCC cells transfected with different si-RNAs filtered from 16 N-glycosyltransferases mRNA expression. **c** Representative confocal images of the colocalization of FUT8 with Endoplasmic Reticulum (calreticulin) and Golgi markers (GM130, Giantin, and TGN46) in HN6 and HN30 cells. Scale bar, 10 μm. **d** Immunofluorescence co-distribution of SEMA7A with LCA lectin in HN6 (up) and HN30 (down) cells treated with si-NC, si-FUT8 and 2F-Fuc. Scale bar, 10 μm. **e** Flow cytometry measuring the binding activity of SEMA7A with Plexin C1 in HNSCC cells transfected with si-NC and si-FUT8, followed by SEMA7A-WT and 5NQ. **f** Cell viability detection of HN6 (up) and HN30 (down) cells transfected with si-NC and si-FUT8 at designed timepoints (0, 1, and 3 days). **g** Representative IHC images with diverse FUT8 intensities (negative, low, and high) staining in tissue microarray from HNSCC tumors. Scale bar, 50 μm. **h** Kaplan–Meier plots of the overall survival of HNSCC patients stratified by the IHC score of FUT8 (*p* value was calculated using the log-rank test). (Data were shown as mean ± SEM, *P ≤ 0.05, **P ≤ 0.01, ***P ≤ 0.001, ****P ≤ 0.000 1)

### Fucosyltransferase FUT8 catalyzes aberrant SEMA7A N-glycosylation

Subsequently, we sought to identify the underlying mechanism that regulates the aberrant N-glycosylation of SEMA7A. The genes encoding sixteen glycotransferases responsible for N-glycosylation were obtained, and their transcript levels were analyzed in different HNSCC cell lines.^11^ Among these genes, FUT8, ST3GLA1, B3GAT1, B3GAT2, GCNT3, and MAGT3 were significantly upregulated in both HN6 and HN30 cells compared with normal epithelia (Fig. 5a). Based on this, we then validated the determined role of these six glycotransferases in the N-glycosylation of SEMA7A by using lectin blotting. The successful synthesis of the siRNAs and their knockdown efficiency in both cell lines were verified (Supplementary Fig. 6). First, knockdown of these glycotransferases did not affect the total protein expression level of SEMA7A. By using LCA, Con A and SNA lectin pull-down assays, we found an obvious reduction in SEMA7A enrichment in si-FUT8, si-ST3GLA1, and si-GCNT3 cells. Regarding PHA-L lectin enrichment, a decrease in SEMA7A enrichment was found in si-FUT8, si-B3GAT1, si-B3GAT2, and si-GCNT3 cells. LCA, representing core fucosylation, was the most abundant lectin in both HN6 and HN30 calls, as determined by flow cytometry (Fig. 5b). Therefore, we speculated that FUT8 may be the major catalyzer responsible for the aberrant glycosylation of SEMA7A.

**Fig. 6:**
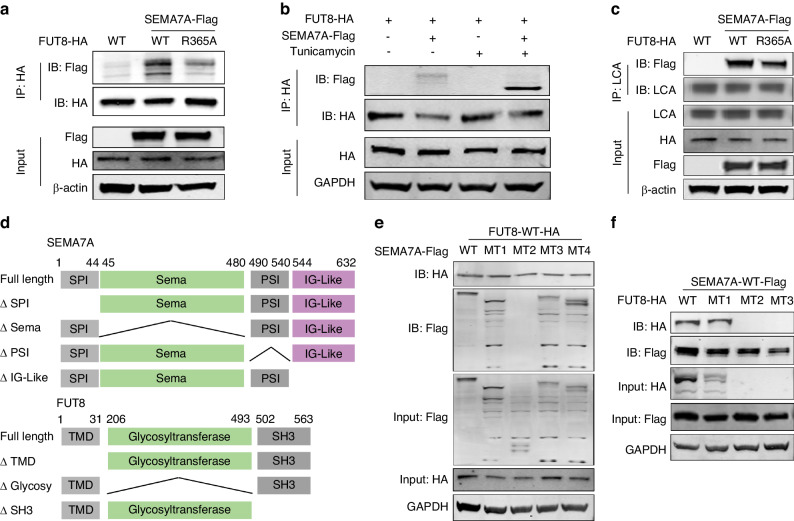
FUT8 mediated abnormal glycosylation of SEMA7A through FUT8-SEMA7A proteins interaction. **a** Total (Input) or immunoprecipitated (IP) lysates from HN6 cells transfected with Flag-tagged SEMA7A-WT together with HA-tagged FUT8-WT or FUT8-R365A were immunoblotted using the indicated antibodies. **b** IP analysis of the interaction of exogenous SEMA7A-WT-Flag with FUT8-WT-HA in the presence or absence of tunicamycin for 24 h. **c** LCA affinity of whole-cell lysate of HN6 cells overexpressed with SEMA7A-WT-Flag as well as FUT8-WT-HA or FUT8-R365A-HA by western blot with anti-SEMA7A. **d** Schematic diagram of truncated fragments of SEMA7A and FUT8 according to the different functional domains. **e**, **f** HN6 cells were transiently transfected with truncated HA-tagged FUT8 (**e**) and Flag-tagged SEMA7A (**f**) mutations, followed by immunoprecipitation with anti-HA and Flag beads and subsequent immunoblot analysis with anti-Flag and HA

FUT8 is mainly distributed in the Golgi, as evidenced by the colocalization of the FUT8 protein with Golgi markers (GM130 for the cis-Golgi, Giantin for intercisternal cross-bridges of the Golgi complex, and TGN46 for the trans-Golgi) but not the ER marker calreticulin (Fig. 5c). Downregulation of FUT8 had no significant influence on the transcript and protein levels of SEMA7A (Supplementary Fig. 7). Subsequently, we investigated whether FUT8 knockdown has a determining role in the core fucosylation of SEMA7A. 2F-Fuc is a typical inhibitor of core fucosylation and significantly abolishes LCA binding to SEMA7A. Similarly, FUT8 knockdown also dramatically decreased the fluorescence intensity of LCA, indicating that FUT8 is necessary for the core fucosylation of SEMA7A (Fig. 5d). In addition, downregulation of FUT8 markedly attenuated the binding of SEMA7A-WT to Plexin C1 (si-NC/SEMA7A-WT, 21 713 ± 940; si-FUT8/SEMA7A-WT, 16 651 ± 1108). However, this alteration in binding was not observed for SEMA7A-5NQ/Plexin C1 (si-NC/SEMA7A-5NQ, 11 252 ± 894; si-FUT8/SEMA7A-5NQ, 11 066 ± 688) (Fig. 5e, Supplementary Fig. 8). Then, we tried to clarify whether overexpression of FUT8 in tumors promotes oncogenesis. Knockdown of FUT8 in tumor cells significantly abrogated in vitro proliferation (Fig. 5f). Through HNSCC tissue microarray analysis, we further confirmed that higher expression of FUT8 in tumor cells (high: positive ratio ≥ 10%; low: positive ratio < 10%) was intimately associated with poorer prognosis (Fig. 5g, h).

**Fig. 7:**
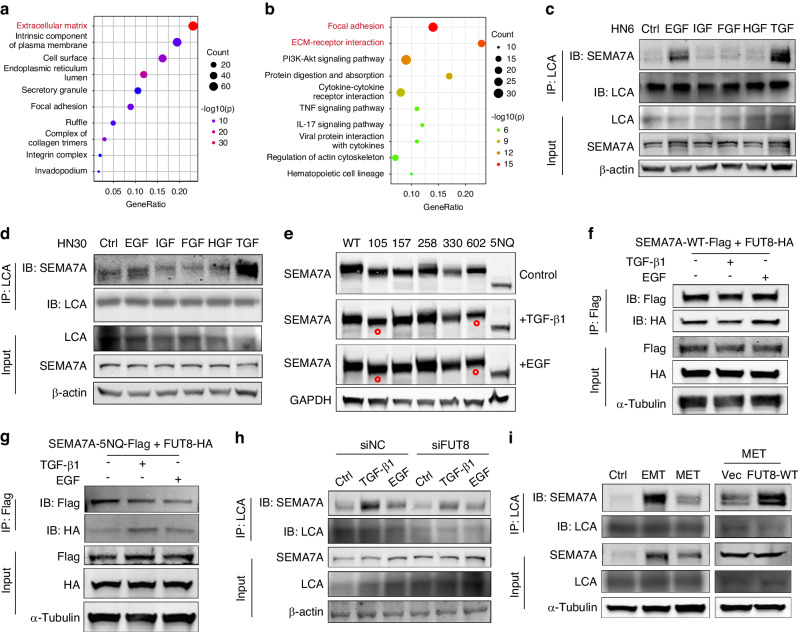
EGF and TGF-β1 signaling induce N-glycosylation of SEMA7A. **a**, **b** GO and KEGG analyses of the HNSCC tumors from TCGA-HNSCC stratified according to the different abundance of SEMA7A. **c**, **d** Western blot analysis of the binding affinity of SEMA7A with LCA in HN6 (**c**) and HN30 (**d**) cells treated with EGF, IGF, FGF, HGF, and TGF-β1 for overnight. **e** Glycosylation recovery of HN6 cells expressing with wild type SEMA7A or its N-glycosylation site mutants in the presence or absence of TGF-β1 and EGF. **f**, **g** The interaction activity of exogenous SEMA7A-WT-Flag (**f**) and SEMA7A-5NQ-Flag (**g**) with FUT8-WT-HA in the presence or absence of TGF-β1 and EGF by using IP and subsequent western blot analysis. **h** The influence of FUT8 intervention on the EGF and TGF-β1 signaling mediated N-glycosylation of SEMA7A by using lectin enrichment and western blot. **i** The determined role of EMT and MET events on the binding affinity of LCA with SEMA7A in lectin blot

**Fig. 8:**
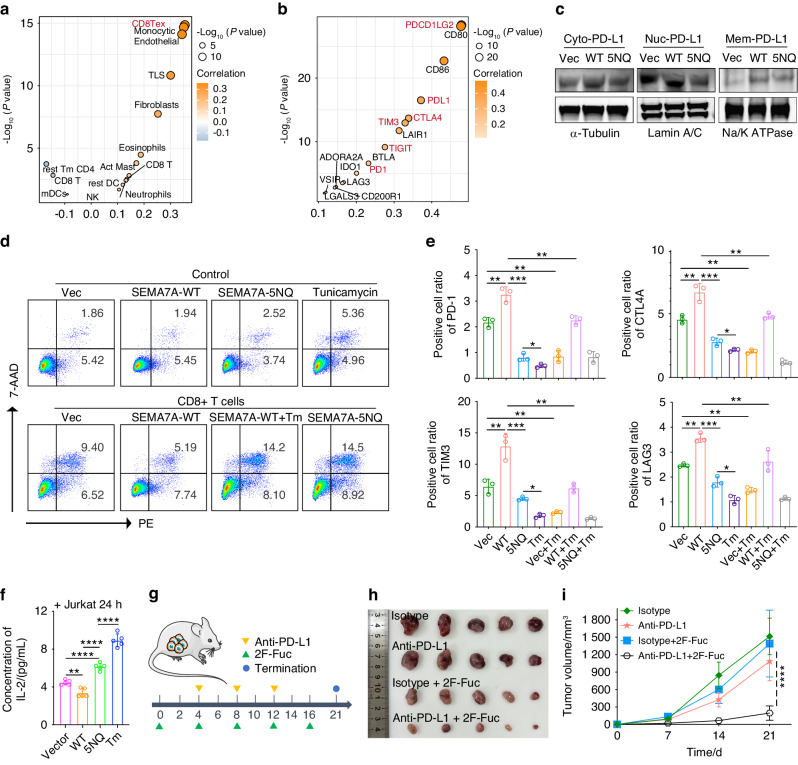
Deglycosylation of SEMA7A contributes to the EGFR targeted and immune therapy. **a**, **b** Correlation analysis of SEMA7A abundance with tumor infiltrate lymphocytes (**a**) and checkpoint expression (**b**). **c** The biological influence of SEMA7A N-glycosylation on PD-L1 protein expression in membrane, cytoplasm and nucleus using cell fractionation and subsequent immunoblot analysis. **d** The indicated HN6-SEMA7A KD cells were transiently transfected with SEMA7A-WT and 5NQ plasmids in the presence of tunicamycin or not, then cocultured with CD3/CD28-activated human CD8^+^ T-lymphocyte cells. Representative plots of the tumor apoptosis were measured by FACS. **e** Detection of the checkpoints (PD-1, CTLA4, TIM3, and LAG3) expression in human CD8^+^ T-lymphocyte cells cocultured with tumor cells transfected with SEMA7A-WT and 5NQ in the presence or absence of tunicamycin by flow cytometry. **f** Elisa measuring of IL-2 release in Jurkat cells cocultured with HNSCC cells overexpressed with SEMA7A-WT and 5NQ or pre-treated with tunicamycin. **g** The therapeutic protocol of tumor growth of Sema7a-WT re-expressed SCC7-Sema7a-KD cells in C3H mice following treatment with 2F-Fuc and anti-PD-L1 antibody. **h**, **i** In vivo tumor growth in four groups with different treatments presented as tumor specimens (**h**) and growth curve (**i**). (Data were shown as mean ± SEM, ** *P* ≤ 0.01, *** *P* ≤ 0.001, ****P ≤ 0.000 1)

### Interaction of FUT8 with SEMA7A during aberrant glycosylation

We speculated that FUT8 may directly interact with SEMA7A during glycan modification. WT-HA-tag FUT8 and R365A-HA-tag FUT8 (inactive mutant) plasmids were constructed and co-transfected with the SEMA7A-WT-Flag-tag plasmid into tumor cells. Immunoprecipitation (IP) of HA verified the direct binding of FUT8 to glycosylated SEMA7A. However, FUT8-R365A showed decreased binding capacity compared with FUT8-WT, indicating that the active form of FUT8 was indispensable for its catalyzing role in glycosylation (Fig. 6a). Ultimately, we tried to identify whether the glycosylation status of SEMA7A was necessary for its protein‒protein interaction (PPI). In the presence of tunicamycin, deglycosylated SEMA7A exhibited a greater binding ability than the glycosylated form (Fig. 6b). As shown above, eradication of glycosylation may enhance antigen exposure and detection, which may account for the increased interaction between FUT8 and deglycosylated SEMA7A. To delineate whether the functional status of FUT8 was associated with SEMA7A glycosylation, we evaluated LCA enrichment, which showed a significant reduction in the binding of LCA to SEMA7A in FUT8-R365A cells compared with FUT8-WT cells, indicating that the active form of FUT8 was necessary for glycan modification of SEMA7A (Fig. 6c).

STX18 is a SNARE protein that is involved in membrane trafficking between the ER and Golgi.^32^ Through PPI network analysis, we found that STX18 is a candidate binding partner for FUT8. This finding led us to speculate that STX18 may act as a trafficking protein during FUT8-mediated SEMA7A glycosylation. STX18 knockdown has no determined role in SEMA7A protein expression and glycosylation (Supplementary Fig. 9a). By co-transfection with Myc-tag STX18, HA-tag FUT8, and Flag-tag SEMA7A, co-IP verified pairwise interactions among the three proteins (Supplementary Fig. 9b). These data indicated that STX18 is a transport protein for the FUT8/SEMA7A complex. To further investigate the influence of protein synthesis and transport on the pairwise interactions of these three proteins, co-transfected tumor cells were incubated with CHX (inhibitor of protein synthesis) and brefeldin A (BFA, inhibitor of protein trafficking), with the finding that BFA significantly abrogated the interactions between STX18 and SEMA7A, FUT8 and STX18 and between FUT8 and SEMA7A. However, inhibition of protein synthesis only affected the binding of STX18 and SEMA7A (Supplementary Fig. 9c–e). These Co-IP data demonstrated that protein transport is a necessary process for FUT8-mediated SEMA7A glycosylation.

**Fig. 9:**
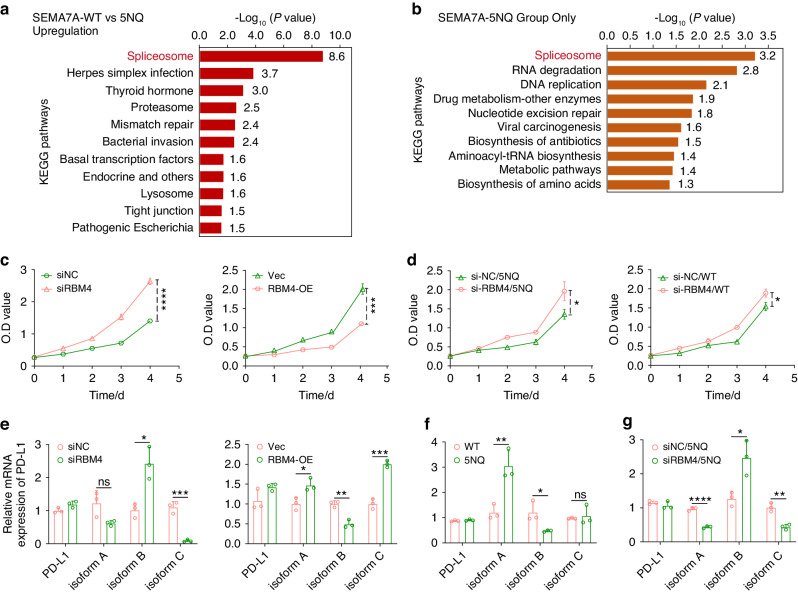
N-glycosylation of SEMA7A induced RBM4 upregulation is associated with PD-L1 alternative splicing. **a**, **b** Proteomic analysis of the protein lysates from HN6 cells overexpressed with SEMA7A-WT and 5NQ (*n* = 3). Functional enrichment of differential expressed proteins between two groups were subjected to KEGG pathway analysis. **c** In vitro proliferation of si-NC/si-RBM4 (left) and Vector/RBM4-OE plasmids (right) transfected HNSCC cells using CCK8 assay at indicated time interval. **d** Cell proliferation measuring of HNSCC cells sequentially transfected with SEMA7A-WT/5NQ and si-NC/si-RBM4. **e** Detection of PD-L1 alternative splicing in HNSCC cells following RBM4 intervention. **f** Expression of PD-L1 isoforms in HN6 cells transfected with SEMA7A-WT and 5NQ using real-time PCR. **g** Transcriptive level of PD-L1 isoforms in HN6 cells sequentially transfected with SEMA7A- 5NQ and si-NC/si-RBM4. (Data were shown as mean ± SEM, *P ≤ 0.05,**P ≤ 0.01, ***P ≤ 0.001, ****P ≤ 0.000 1)

SEMA7A is composed of SPI, Sema, PSI and IG-like domains, and FUT8 includes TM, glycosyltransferase and SH3 domains.^33,34^ To pinpoint the specific interaction domain between FUT8 and SEMA7A, we generated 4 Flag-tagged SEMA7A truncation fragments and 3 HA-tagged FUT8 truncation fragments (Fig. 6d). HN6 cells were cotransfected with SEMA7A full-length/4 fragments with FUT8-WT or with FUT8 full-length/4 fragments with SEMA7A-WT and subsequently analyzed by Flag or HA enrichment and further immunoblot analysis. A missing band was found for the fragment with deletion of the Sema domain after HA enrichment, and bands were absent in the Δ Glycosy and Δ SH3 fragments following anti-Flag precipitation (Fig. 6e, f). These data supported the concept that the Sema domain in SEMA7A and the glycosyltransferase/SH3 domains in FUT8 are the specific domains necessary for this protein‒protein interaction. The subcellular distribution of the truncated FUT8 fragments, as evaluated by confocal microscopy, further verified the glycosyltransferase/SH3 domain specificity, as evidenced by the different localization statuses of the truncations with ER and Golgi markers (Supplementary Fig. 10a). In addition, the recognition of SEMA7A by Plexin C1 was distinct from that of SEMA7A by FUT8. Flow cytometry showed that the Sema, PSI and IG-like domains are all indispensable for the formation of the SEMA7A/Plexin C1 complex (Supplementary Fig. 10b).

**Fig. 10:**
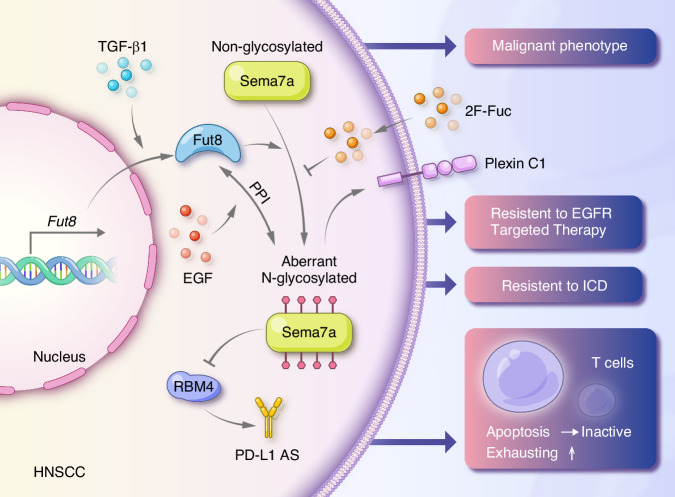
Molecular mechanism of FUT8 mediated aberrant SEMA7A glycosylation and its promotive role in HNSCC progression

### **EGF and TGF-β1 induce SEMA7A glycosylationviadifferent mechanisms**

Through GO and KEGG analyses of the HNSCC database from the cancer genome atlas (TCGA-HNSCC), we discovered that a high abundance of SEMA7A was associated with extracellular matrix (ECM) organization and ECM receptor interaction, indicating that SEMA7A participated in tumor microenvironment remodeling (Fig. 7a, b). To identify the ECM-related cytokines that govern SEMA7A N-glycosylation, we treated HNSCC cell lines with several growth factors, including transforming growth factor (TGF-β1), epidermal growth factor (EGF), fibroblast growth factor (FGF), insulin-like growth factor (IGF) and hepatocyte growth factor (FGF). After LCA enrichment, dramatic accumulation of SEMA7A was found only in TGF-β1- and EGF-treated HN6 and HN30 cells, suggesting the inducing effect of these two cytokines on the N-glycosylation of SEMA7A (Fig. 7c, d). Therefore, we investigated whether TGF-β1 and EGF mediate SEMA7A glycosylation via a similar molecular mechanism. First, we examined the reglycosylation of individual mutation sites following growth factor incubation and found that compared with TGF-β1 treatment, EGF treatment resulted in an increased abundance of SEMA7A glycosylated at residues 105 and 602 (Fig. 7e). Next, we investigated the determining role of growth factors in the interaction between SEMA7A and FUT8. Co-IP analysis showed that compared with TGF-β1, EGF significantly increased the binding activity of SEMA7A-WT toward FUT8, an effect that failed to be repeated for SEMA7A-5NQ (Fig. 7f, g). Furthermore, we tried to elucidate whether FUT8 intervention has a similar effect on TGF-β1- and EGF-induced SEMA7A glycosylation. HN6 cells were treated with si-NC and si-FUT8 prior to incubation with growth factors, and the binding of the SEMA7A protein with LCA then was detected. Interestingly, FUT8 knockdown substantially attenuated the core fucosylation of SEMA7A induced by TGF-β1 but not that induced by EGF (Fig. 7h).

Subsequently, we sought to delineate the association of SEMA7A glycosylation with TGF-β1-induced epithelial-mesenchymal transition (EMT). Through EMT and mesenchymal-epithelial transition (MET) model establishment (Supplementary Fig. 11a), we determined that the binding of LCA to SEMA7A was altered in tandem with the EMT and MET processes (Fig. 7i). Furthermore, during MET, FUT8 overexpression alleviated the deglycosylation of SEMA7A (Fig. 7i). These findings indicated that TGF-β1-mediated SEMA7A glycosylation was regulated by FUT8 in the EMT process. We next investigated the role of SEMA7A and FUT8 in EMT through siRNA and plasmid introduction followed by TGF-β1 stimulation. Surprisingly, the alteration of SEMA7A glycosylation and FUT8 expression had no obvious influence on the EMT process (Supplementary Fig. 11b, c). These results indicated that FUT8-mediated SEMA7A glycosylation was regulated by EMT but not involved in the regulation of EMT. Taken together, these findings indicated that TGF-β1 mediates SEMA7A glycosylation mainly through EMT in a manner mediated by FUT8, while EGF mediates glycosylation mainly by increasing the binding activity of SEMA7A toward FUT8.

### N-glycosylation of SEMA7A mediates the efficacy of EGFR targeting and immunotherapy

EGF signaling is one of the main inducers of SEMA7A glycosylation, and this observation prompted us to hypothesize that inhibition of SEMA7A glycosylation may enhance the efficacy of EGFR-targeted therapy. To this end, we first tested the activation of EGFR signaling in SEMA7A-WT/5NQ cells following EGF incubation. The phosphorylation of ERK1/2, STAT3 and EGFR was dramatically attenuated in SEMA7A-5NQ cells, indicating that SEMA7A glycosylation has a determining role in the transduction of EGFR signaling (Supplementary Fig. 12a). Subsequently, HNSCC cell lines were treated with EGFR inhibitors (gefitinib and erlotinib), and SEMA7A-5NQ cells were found to be more sensitive to EGFR-targeted therapy, as evidenced by the elevated cleaved Caspase-3 activity and decreased cell proliferation rate (Supplementary Fig. 12b, c).

We further investigated the association of SEMA7A with the immunosuppressive microenvironment. Through TCGA-HNSCC data analysis, we proved that overexpression of SEMA7A was positively correlated with elevation of immune checkpoint (PDCD1LG2, PD-L1, CTLA4, TIM3, TIGIT, and PD-1) expression, a decrease in activated CD8^+^ T cells and increased infiltration of exhausted CD8^+^ T cells (Fig. 8a, b). Inspired by these findings, we detected PD-L1 expression in tumor cells by cell fractionation and found that deglycosylation of SEMA7A significantly reduced the PD-L1 levels in the cytoplasm and nucleus but not the cytomembrane (Fig. 8c). Subsequently, we isolated CD8^+^ T cells from peripheral blood and then cocultured them with SEMA7A-WT/5NQ tumor cells and tunicamycin. Flow cytometry showed a higher proportion of apoptotic cells in the SEMA7A-5NQ group, an observation identical to that in tunicamycin-treated cells, indicating that cells with deglycosylation of SEMA7A were more susceptible to CD8^+^ T cell-mediated cytotoxicity (Fig. 8d). Next, we investigated the immunosuppressive effect of SEMA7A glycosylation on CD8^+^ T cells. Aberrant glycosylation of SEMA7A resulted in a higher proportion of exhausted CD8^+^ T cells than did SEMA7A-5NQ, as characterized by the distinct increases in the PD-1, LAG3, CTLA4, and TIM3 levels in T cells (Fig. 8e). In addition, deglycosylation of SEMA7A may partially rescue the cytotoxicity of Jurkat cells, as evidenced by increased IL-2 secretion (Fig. 8f). Finally, we investigated the antitumor efficiency of 2-fluoro-L-fucose (2F-Fuc, a classical inhibitor of cellular core fucosylation) treatment combined with anti-PD-L1 immunotherapy in C3H (immunocompetent) mice, attempting to verify whether blocking the core fucosylation of SEMA7A with 2F-Fuc could improve the outcome of immunotherapy. In C3H mice bearing Sema7a-5NQ-re-expressing SCC7-Sema7a-KO xenograft tumors, there was no combinatorial effect when the tumors were treated with 2F-Fuc and anti-PD-L1 antibodies (data not shown). However, in C3H mice bearing Sema7a -WT-re-expressing SCC7- Sema7a-KO xenograft tumors, the combined treatment with 2F-Fuc and anti-PD-L1 antibodies conspicuously suppressed tumor growth and expansion, as confirmed by the tumor observation and growth curves of the xenograft tumors (Fig. 8g–i). Taken together, these findings indicated that inhibition of SEMA7A glycosylation enhances the efficacy of T-cell-mediated immunotherapy.

Here, we also evaluated the correlation of SEMA7A glycosylation with immunogenic cell death (ICD), which can be induced by doxorubicin (DOX). The typical features of ICD are increased calreticulin (CRT) translocation, elevated autophagy and decreased nuclear HMGB1. In our data, SEMA7A-5NQ and tunicamycin significantly augmented CRT expression and attenuated HMGB1 nuclear accumulation (Supplementary Fig. 13a, b). Moreover, SEMA7A-5NQ cells were more sensitive to DOX therapy, as evidenced by the decreased cell viability (Supplementary Fig. 13c). These results suggested that deglycosylation of SEMA7A markedly enhanced DOX’s activity in ICD induction.

### RBM4 upregulation mediated by N-glycosylation of SEMA7A is associated with PD-L1 alternative splicing

We finally sought to explore downstream signaling in cells with different statuses of SEMA7A N-glycosylation. After transfection with SEMA7A-WT/5NQ, HN6 cells were then subjected to protein extraction and subsequent mass spectrometry and proteomics analysis. KEGG pathway analysis on the basis of differentially expressed proteins (DEPs) showed that the spliceosome ranked at the top in both the mass spectrometry and proteomics data (Fig. 9a, b). By filtering of the list of spliceosome-related DEPs, we found an interesting protein, RBM4, RNA-binding motif 4, a splicing regulator mediating alternative splicing programs (Supplementary Table 2).^35–37^ The elevation of RBM4 expression induced by SEMA7A-5NQ was further verified in HN30 cells (Supplementary Fig. 14a). We then tried to uncover the molecular mechanism of SEMA7A-5NQ-triggered upregulation of RBM4. Compared to that in the control group, SEMA7A-WT inhibited RBM4 transcription, while SEMA7A-5NQ partially rescued the mRNA expression of this splicing factor (Supplementary Fig. 14b). In addition, we observed a lower turnover rate of the RBM4 protein in SEMA7A-5NQ-transfected cells at 0, 4, 12, and 24 h than in SEMA7A-WT cells (Supplementary Fig. 14c). All of these results indicated that SEMA7A-5NQ upregulated RBM4 expression by augmenting its mRNA transcription and retarding its protein degradation.

RBM4 has been reported to be involved in cell proliferation. To delineate the functional role of RBM4 in the tumor microenvironment, we performed gain and loss of function experiments. In vitro cell viability analysis demonstrated that RBM4 has a negative regulatory role in HNSCC cells (Fig. 9c). Then, we sought to determine whether the SEMA7A-5NQ-mediated cell growth inhibition was regulated by RBM4. Tumor cells were cotransfected with si-RBM4/RBM4-OE and SEMA7A-WT/5NQ and were then subjected to a CCK8 assay. RBM4 was the main regulator that participated in cell growth under SEMA7A deglycosylation conditions (SEMA7A-5NQ). In regard to glycosylation status, RBM4 only partially accounts for the regulatory effects on cell proliferation (Fig. 9d, Supplementary Fig. 15a). However, RBM4 knockdown rescued the migration capability in both the SEMA7A-WT and 5NQ groups (Supplementary Fig. 15b). These data indicated that SEMA7A-WT and 5NQ control cell viability through distinct signaling pathways.

As RBM4 is a splicing factor, to find its transcriptome targets, we performed ribonucleoprotein immunoprecipitation (RIP) combined with high-throughput RNA-Seq (RIP-Seq), a method used to detect protein‒RNA interactions in living cells. After RIP-Seq, a total of 13848 transcripts were identified as potential targets for alternative splicing (Supplementary Data). Among these candidate transcripts, alternative splicing (AS) of PD-L1 strongly attracted our interest. PD-L1 pre-mRNA can produce three variants (isoforms A, B, and C) by AS, according to the US National Center for Biotechnology Information (NCBI) database. Distinct roles of these PD-L1 alternative splicing isoforms have been elucidated in colorectal cancer.^38–40^ This prompted us to test whether RBM4 was involved in the AS of PD-L1 in HNSCC cell lines. RBM4 intervention (gain and loss) had no influence on the mRNA expression of full-length PD-L1 but altered the transcript levels of isoforms A, B, and C. Specifically, in RBM4-deficient cells, isoform B expression was increased, with concomitant attenuation of isoform A and C transcription. The opposite regulatory trend was observed in RBM4-overexpressing cells, suggesting that RBM4 contributed to the expression of PD-L1 splice isoforms (Fig. 9e). Next, we investigated whether the glycosylation status of SEMA7A is correlated with the AS of PD-L1. As shown in Fig. 9f, transfection of cells with SEMA7A-5NQ induced expression of diverse PD-L1 isoforms, in line with the trend observed in RBM4-upregulated cells. Subsequently, we attempted to answer the question of whether the glycosylation status-dependent AS of PD-L1 was mediated through RBM4. To this end, we sequentially transfected HNSCC cells with SEMA7A-WT/5NQ vectors and siRBM4 and observed that knockdown of RBM4 did not contribute to the AS of PD-L1 in SEMA7A-WT cells but significantly affected the SEMA7A-5NQ-mediated alteration in PD-L1 isoform transcription, indicating that deglycosylation of SEMA7A promoted PD-L1 AS isoform switching through upregulation of RBM4 (Fig. 9g, Supplementary Fig. 15c).

## Discussion

The current investigation focused on exploring the underlying mechanism of aberrant glycosylation of SEMA7A in HNSCC and its role in tumor progression, EGFR-targeted therapy, immunotherapy, and splicing regulation. In the present study, we first delineated that both overexpression and glycosylation of SEMA7A promoted HNSCC progression. The in-depth analysis demonstrated that FUT8 catalyzed core fucosylation of N-glycans in SEMA7A, which contributed to aberrant SEMA7A glycosylation in HNSCC. More specifically, FUT8, SEMA7A, and STX18 formed a functional complex trafficking from the ER to the Golgi during glycan biosynthesis. Furthermore, FUT8-mediated SEMA7A glycosylation is mainly induced by TGF-β1 and EGF via different regulatory mechanisms in the TME. Finally, targeting SEMA7A glycosylation enhanced the sensitivity of HNSCC cells to EGFR-targeted therapy, CD8^+^ T-cell-mediated immunotherapy and DOX-induced ICD.

Aberrant glycosylation is the most abundant PTM pattern and is deemed the hallmark of malignancies. Different N-glycans are added, trimmed and modified in the process of protein trafficking from the ER to Golgi compartments.^41^ Aberrant glycosyltransferase expression in tumors always results in a variety of glycoforms and structures related to a specific peptide sequence, which may lead to functional alterations in protein subcellular distribution, protein stability, cellular viability control, and receptor‒ligand interactions. Aberrant glycosylation in the TME has been associated with ECM remodeling, cellular interactions, antigen-antibody recognition, inflammation, and immune surveillance and responses.^2^ A highly glycosylated protein, SEMA7A is involved in angiogenesis, liver fibrosis, neurogenesis, dendritic cell migration, and breast and pulmonary cancer progression.^19,20,26,27,29^ However, the functional role of SEMA7A glycosylation has not been reported, especially in tumor progression. In addition, the mechanisms for the maintenance of high glycosylated SEMA7A levels in HNSCC cells are still elusive. In our data, core fucosylation characterized by LCA was the most abundant type of N-glycan modification of SEMA7A in HN6 and HN30 cells. We found that the fucosyltransferase FUT8 was responsible for the N-glycosylation of SEMA7A in HNSCC cells. Via its catalytic addition of fucose to the innermost GlcNAc residue of an N-linked glycan, FUT8 is considered the only glycotransferase responsible for α1,6-linked (core) fucosylation. Transactivated by β-Catenin/LEF-1 signaling, FUT8 overexpression enhances non-small cell lung cancer growth and metastasis.^42^ FUT8 is also a driver of melanoma metastasis by regulating the glycosylation and stability of L1CAM.^43^ In addition, FUT8-mediated aberrant N-glycosylation of B7H3 suppresses the immune response in triple-negative breast cancer,^15^ and FUT8-driven glycosylation of PD-L2 promotes immune evasion and predicts anti-EGFR efficacy.^16^ Our data also showed that FUT8-mediated SEMA7A glycosylation was associated with cell proliferation, CD8^+^ T-cell functional suppression, EGFR signaling activation, and PD-L1 splicing. During protein synthesis and subsequent glycosylation, FUT8 executes its catalytic function by directly binding to SEMA7A, which is transported by STX18 from the ER to the Golgi. The protein trafficking inhibitor BFA significantly abrogated this protein‒protein interaction. FUT8 was also found to directly interact with B7H3 during glycan modification.^15^ In addition, we first demonstrated that the glycotransferase and SH3 domains of FUT8 were indispensable for their binding to the Sema domain of SEMA7A. Taken together, these data indicated that the formation of the FUT8/SEMA7A complex is necessary for the glycosylation of SEMA7A.

SEMA7A also participates in the remodeling of the immune microenvironment. TCGA-HNSCC bioinformatic analysis showed that SEMA7A is coexpressed with checkpoints, such as PD-L1, CTLA4, TIM3, TIGIT, and PD-1, which indicate an immunosuppressive state. Furthermore, we investigated immune cell infiltration in tumors with higher SEMA7A expression and found that SEMA7A expression was positively correlated with Treg and exhausted CD8^+^ T-cell infiltration and negatively associated with active CD8^+^ T-cell accumulation. These discoveries indicated that SEMA7A may have an intimate relationship with the immunosuppressive microenvironment. Our data proved the regulatory role of glycosylated SEMA7A in the increased proportion of CD8^+^ Tex cells and reduced cytotoxicity. In contrast, cells with non-glycosylated SEMA7A were more susceptible to attack from CD8^+^ T cells and DOX-induced immunogenic cell death, indicating that targeting glycosylated SEMA7A is a promising intervention to enhance the therapeutic efficacy of PD-1 blockade. This was in line with the findings that blockade of B7H3 core fucosylation enhances the antitumor immune response,^15^ that PD-L2 glycosylation negatively modulates antitumor immunity,^16^ that glycosylation and stabilization of programmed death ligand-1 suppresses T-cell activity,^8^ that removal of N-linked glycosylation enhances PD-L1 detection and predicts anti-PD-1/PD-L1 therapeutic efficacy,^12^ and that triple-negative breast cancer cells can be eradicated by targeting glycosylated PD-L1.^11^

Since EGF is a main inducer of SEMA7A glycosylation in the TME, we investigated the contribution of glycosylated SEMA7A to EGFR signaling and targeted therapy. Our data revealed that deglycosylation of SEMA7A markedly blocked the transduction of EGFR signaling and augmented the sensitivity of cells to the targeted therapeutic agents gefitinib and erlotinib. This phenomenon was identical to the finding that removal of PD-L1 and PD-L2 glycosylation enhanced the antitumor efficacy of gefitinib and cetuximab therapies, respectively.^8,16^ Carcinoembryonic antigen-related cell adhesion molecule 6 (CEACAM6) interacts with the EGFR receptor and then activates signal transduction via MGAT5-mediated N-glycosylation.^44^ In addition, glycosylation of the EGFR receptor mediated by GALNT2 promotes oral cancer cell migration and invasion.^17^ Taken together, these observations indicate that EGFR signaling is intimately correlated with the glycosylation status of tumor-associated proteins. Deglycosylation is thus a novel option for synergism with EGFR targeted therapy.

In summary, our results identified the underlying mechanism of aberrant N-glycosylation of SEMA7A and its promotive role in the oncogenesis of HNSCC. Aberrant glycosylation in SEMA7A could be induced by cytokines in the tumor microenvironment, such as EGF and TGF-β1, through distinct molecular pathways. Deglycosylation of SEMA7A augmented the sensitivity of HNSCC to EGFR-targeted therapy and attenuated immunosuppression, thus facilitating the response to anti-PD-L1-based immunotherapy (Fig. 10). Our findings provide a rationale for combining SEMA7A glycosylation blockade with EGFR targeted therapy and immunotherapy.

## Materials and methods

### RNA sequencing and data analysis

Total RNA was extracted from the indicated cell samples with a RNeasy Plus Mini Kit (Qiagen) according to the manufacturer’s protocol. RNA was subjected to RNA-Seq analysis on a BGISEQ-500 system by Beijing Genomics Institute (BGI), China. In addition, the RNA was sheared and reverse transcribed through random primers to obtain the cDNA for library construction. Subsequently, sequencing was performed on the prepared library. All the generated raw sequencing reads were filtered to obtain clean reads, which were stored in FASTQ format. Bowtie2 and HISAT were used to map clean reads to reference genes and genomes, respectively. RSEM was used to quantify gene expression levels (FPKM). The NOISeq method was used to screen out differentially expressed genes between two groups with a fold change ≥ 2 and divergence probability ≥0.8. Gene Ontology (GO) and pathway annotation and enrichment analyses were performed based on the Gene Ontology Database (http://www.geneontology.org/) and KEGG pathway database (http://www.genome.jp/kegg/), respectively.

### qRT–PCR assays

For quantitative reverse transcription PCR (qRT‒PCR) assays, total RNA was extracted from HNSCC cells using the Axygen Total RNA Isolation Kit and further reverse transcribed using the PrimeScript ™ RT Reagent Kit with gDNA Eraser (Takara, Japan). Then, synthesized cDNA was subjected to real-time PCR in the presence of a One Step SYBR^@^ PrimeScript^@^ PCR Kit (Takara, Japan) in an Applied Biosystems StepOne™ machine (Invitrogen, USA). The differential expression of target genes was evaluated and quantified using the formula 2^−ΔΔCT^, with target gene expression levels normalized to that of the housekeeping gene GAPDH. The designed primer sequences used in the current research are listed in Supplementary Table 3.

### TCGA bioinformatics analysis

The transcriptomic data were acquired from TCGA HNSCC samples with the associated patient clinical characteristics, therapeutic regimens, corresponding responses, follow-up data, RNA-Seq data and somatic mutation data. The HNSCC RNA sequencing data (FPKM values) were downloaded via the R package TCGA biolinks and were subsequently transformed into transcripts per kilobase million (TPM) values.

We retrieved the standard data from the TCGA database after log2 scaling and RMA normalization. Then, we focused on the genes and signatures that were coexpressed with SEMA7A. Genes whose expression was positively and negatively correlated with SEMA7A expression were then subjected to Gene Ontology (GO) and Kyoto Encyclopedia of Genes and Genomes (KEGG) analyses using the R package clusterProfiler (v3.14.3). The infiltration status of different immune cell types in the TME of HNSCC was quantified and compared between the two groups by using two independent algorithms: the “CIBERSORT” R package and MCP-counter.

### Antibodies and chemicals

Information about the antibodies, chemicals and recombinant proteins used in the current investigation are listed in Supplementary Table 4.

### Cell culture

All cell lines, including HUVECs, HNSCC cell lines (HN4, HN6, HN30, Cal-27, SCC7, SCC9), and immune cell lines (primary CD8^+^ T cells and Jurkat T lymphocytes), were cultured at 37 °C and 5% CO_2_. HUVECs and HNSCC cell lines were maintained in Dulbecco’s modified Eagle’s medium (DMEM)/high glucose with a combination of 10% fetal bovine serum (FBS) and 1% penicillin‒streptomycin. Immune cells were grown in ImmunoCult™-XF T-Cell Expansion Medium containing IL-2 (1 ng/mL).

### Lentiviruses, plasmids and siRNA construction and transfection

The lentiviral shRNA (2494-PGMLV-SC5 plasmid) constructed for downregulation of human SEMA7A was obtained from Genomeditech (Shanghai, China). After verification of the knockdown efficiency of SEMA7A protein expression in HN6 and HN30 cells, two suitable clones were finally selected. The mature antisense sequences were as follows: 5′-TCAATTGTCATATTGCTA C-3’ (sh-SEMA7A #5) and 5′ -TTGACTCCATCTTTC TTCA-3′ (sh-SEMA7A #6). For stable cell generation, HNSCC cells were infected with viral particles in the presence of polybrene (10 μg/ml), and positive stable clones were then selected with puromycin and further subjected to single-cell dilution cloning.

Human full-length SEMA7A DNA was subcloned into pcDNA3.1 (+) at the HindIII/EcoRI restriction site. With full-length SEMA7A as a template, we constructed individual glycosylated site mutants (N105Q, N157Q, N258Q, N330Q, N602Q), the 3NQ (105, 157, 258) mutant and the 5NQ (105, 157, 258, 330, 602) mutant. Based on this, SEMA7A-WT, 3NQ and 5NQ were further fused to a C-terminal Flag tag. For FUT8 overexpression, full-length FUT8 was also integrated into pcDNA3.1 (+), based on which the FUT8-R365A mutant with a C-terminal HA tag was generated. Similarly, full-length STX18 and RBM4 were generated by using pcDNA3.1 (+) with C-terminal Myc and Flag tags, respectively. All constructs were confirmed by using DNA sequencing. Transfection was conducted on cells by using the plasmids, Lipo-3000 and P3000 at a volume ratio of 1:3:2.

For transient knockdown of target genes, we designed and synthesized siRNAs targeting 6 glycotransferases (FUT8, ST3GLA1, B3GAT1, B3GAT2, GCNT3, and MAGT3), STX18, and RBM4. The knockdown efficiency was verified by RT‒PCR. Transfection was conducted on cells by using the siRNA (100 nm) and Lipo-3000 (2 μL/mL).

### Fragment and truncation construction

To identify the interaction domains between SEMA7A and FUT8, we synthesized truncations of SEMA7A and FUT8. SEMA7A is composed of SPI, Sema, PSI, and IG-like domains, and FUT8 includes TM, glycosyltransferase, and SH3 domains. We generated 4 Flag-tagged SEMA7A truncation fragments: ΔSPI, with deletion of amino acids 2-44 and retention of amino acids 45-666; Δ Sema, with deletion of amino acids 45-480 and retention of the remaining sequence; Δ PSI, with removal of amino acids 490-540 and retention of the remaining domains; and Δ IG-like, with deletion of amino acids 544-632. Similarly, we generated 3 HA-tagged truncation fragments of FUT8: Δ TM, with deletion of amino acids 2-31; Δ Glycosyltransferase, with removal of amino acids 206-493; and Δ SH3, with deletion of amino acids 501-575. All of these peptides retained the promoter regions and were verified by DNA sequencing.

### Glycosylation analysis of SEMA7A

To confirm glycosylation of the SEMA7A protein, HNSCC cell lines were incubated with tunicamycin and swainsonine (Sigma‒Aldrich, Germany), and cell lysates were incubated with PNGase F and O-glycosidase (New England BioLabs, Ipswich, MA, USA). LCA modification of SEMA7A was further confirmed through lectin blotting, flow cytometry, and immunofluorescence staining by using Biotinylated Lens Culinaris Agglutinin and DyLight 488 Streptavidin (Vector lab).

### Animal treatment protocol

BALB/c nude mice, Nod- SCID mice, and C3H mice (female, 4-6 weeks) were acquired from Shanghai Jihui Laboratory Animal Care Co., Ltd. and were raised under SPF conditions in the Animal Center of Shanghai Ninth People’s Hospital. Treatment protocols have been reported and were approved by the Ethics Committee of our institution. HN6 cells (3 × 10^6^) transfected with shRNA-SEMA7A, SEMA7A-WT, or SEMA7A-5NQ were injected into the right flanks of BALB/c nude mice to establish the xenograft model for tumorigenesis assays. To evaluate the efficiency of N-glycosylation of SEMA7A in vivo, tumor xenografts were established by injection of 2 × 10^6^ SCC7-SEMA7A KO cells reconstituted with SEMA7A-WT or SEMA7A 5NQ into the flank region of C3H mice. The tumor cells were preincubated with 300 μmol/L 2F-Peracetyl-Fucose (2F-Fuc) or DMSO for 7 days before implantation. After tumor establishment, the animals were randomly divided into four groups: PBS + Isotype (IgG2b), PBS + anti-PD-L1, 2F-Fuc + Isotype, and 2F-Fuc + anti-PD-L1. The mouse anti-PD-L1 antibody (100 μg per mouse) was injected intraperitoneally at the designated timepoints, and 2F-Fuc (3.51 mg/mL in PBS) was given by oral gavage. The tumor volumes were recorded every four days and calculated by the following formula: 0.5 × length (mm) × width (mm). In addition, the body weight and tumor weight were measured during tumorigenesis and after mouse sacrifice.

### T-cell-mediated tumor cell killing assay

CD8^+^ T cells were isolated from human peripheral blood by using the EasySep™ Human CD8^+^ T-Cell Isolation Kit (STEMCELL™ Technologies) and further cultured with ImmunoCult™-XF T-Cell Expansion Medium (STEMCELL™ Technologies) in the presence of human recombinant IL-2 for cell activation. After expansion, CD8^+^ T cells were identified with anti-CD8 and anti-CD3 antibodies. (STEMCELL™ Technologies). Subsequently, active T cells were cocultured with HNSCC cells for 24 h in 12-well plates at a ratio of 5:1 in triplicate. Tumor cells were then subjected to flow cytometry for apoptosis analysis by using an Annexin V-FITC/PI detection kit. After incubation of T cells with tumor cells at a ratio of 1:1 for 24 h, T cells were treated with anti-PD-1, anti-CTLA4, anti-TIGIT and anti-TIM3 antibodies, and the fluorescence intensity of each reagent was detected by using flow cytometry to evaluate the extent of T cell exhaustion.

### Western blot analysis and immunoprecipitation

Cell lysates were obtained by using RIPA buffer (Cat#89901, ThermoFisher Scientific) and protease inhibitor cocktail (Bimake LTD, USA). The protein concentration was determined by a Pierce™ BCA protein assay kit (ThermoFisher Scientific). Samples with 25 μg of protein were separated by SDS‒PAGE, proteins were transferred to PVDF membranes, and the membranes were then incubated with primary and secondary antibodies. To conduct co-IP experiments, cells were lysed using IP Lysis Buffer (Thermo Fisher Scientific) and protease inhibitor. Immunoprecipitation was performed by incubation of the protein suspension (200 μL) with Pierce Protein A/G Magnetic Beads (25 μL) (Thermo Fisher Scientific) and anti-Flag, HA, and Myc magnetic beads (25 μL) (Bimake LTD, USA) overnight at 4 °C. Acquired samples were subsequently subjected to immunoblotting. All PVDF membranes were scanned through an Odyssey infrared imaging system (LI-COR Biosciences, Lincoln, NE, USA). The bands were finally analyzed and quantified by ImageJ software.

### Lectin enrichment and blotting

The antigen samples (200 μL) acquired by IP lysis buffer were combined with 10 μg biotinylated lectin (LCA, SNA, VVL, PHA-L and Con A) (Vector Laboratories) overnight at 4 °C and then incubated with 50 μL Pierce™ Streptavidin magnetic beads (Thermo Fisher Scientific) at RT for 1 h. After washing and elution buffer were used for antigen retrieval, the supernatant containing targeted proteins was then subjected to immunoblot analysis. Proteins were transferred to a PVDF membrane, which was then incubated with a primary antibody (specific for biotinylated lectins or SEMA7A) and a secondary antibody (horseradish peroxidase–streptavidin- or DyLight-conjugated) and finally observed in an infrared imaging system.

### Cell proliferation and migration assays

HNSCC cells were transfected with shRNAs, plasmids and siRNAs. Cells with these genetic interventions were then seeded in 96-well plates at a concentration of 1 000 cells per well and cultured at 37 °C for 5 days. At the designated detection timepoint, the culture medium in each well was replaced by 100 μL fresh DMEM with 10 μL CCK8 and incubated at 37 °C for 2 h. The absorbance of each well was measured by a microplate reader (Tecan Infinite 200) at a wavelength of 450 nm.

For the migration assay, genetically modified cells were seeded into a Transwell chamber and then cultured in 24-well plates for 24 h. Medium with 10% FBS was added to the lower compartments as a chemoattractant. Then, the migrated cells were fixed with 4% paraformaldehyde for 20 min and treated with 0.1% crystal violet for 1 h. The invaded cells in the bottom compartment of the chamber were counted under a microscope in at least 5 randomly selected fields.

### Flow cytometric analysis of lectin enrichment and SEMA7A/Plexin C1 binding

To evaluate the influence of glycosylation on the binding affinity of SEMA7A for Plexin C1, genetically modified HNSCC cells were seeded in 6-well plates and incubated with 2.5 μg/mL recombinant human Plexin C1 with a C-terminal 6-His tag (R&D SYSTEMS) for 24 h. The control group was treated with hIgG.Fc with a 6-His tag. The cells were then collected, washed and incubated with Alexa Fluor® 647‑conjugated Human IgG with a 6-His Tag for 30 min. After final washing with cold PBS, the cells were subjected to analysis by flow cytometry (BD FACSCanto II).

### Cell fractionation

To obtain cytoplasmic, membrane and nuclear proteins, cells in 10 cm dishes were washed and centrifuged at 350 x g for 5 min, and the pellet was then resuspended in 500 μL of 1 x PBS. This suspension of cells was subjected to protein extraction with different constituents of the Cell Fractionation Kit (Cell Signaling Technology) according to the manufacturer’s instructions. Acquired protein samples were used for immunoblot analysis.

### Coculture and IL-2 content measurement

To evaluate the influence of HNSCC cells on T-cell inactivation, tumor cells were coincubated with activated Jurkat T cells in the presence of Human T-Activator CD3/CD28 (STEMCELL™ Technologies) at a ratio of 3:1 (Jurkat: tumor cell). After coculture for 24 h, the supernatant was collected and further subjected to measurement of secreted IL-2 levels according to the manufacturer’s instructions (Human IL-2 ELISA Kits, Thermo Scientific).

### Proteomics study and bioinformatics analysis

The cell pellets were lysed by 8 mol/L urea in 100 mmol/L Tris-HCl (pH 8.5) with protease cocktail (Merck) to denature proteins and subjected to two rounds of sonication on ice to disrupt protein‒protein and DNA‒protein interactions. The protein concentration was measured, and equal amounts of protein were used for tryptic digestion. TCEP [tris(2-carboxyethyl) phosphine, final concentration is 5 mmol/L] (Thermo Scientific) and iodoacetamide (final concentration is 10 mmol/L) (Sigma) for reduction and alkylation, respectively, were added to the solution and incubated at room temperature for 30 minutes. The protein mixture was diluted fourfold and digested with trypsin at a 1:50 ratio (w/w) (Promega) at 37 °C overnight. The tryptic-digested peptide solutions were desalted on MonoSpin™ C18 columns (GL Science), and the eluents were dried in a speed vacuum. The peptide mixtures were finally dissolved in HEPES buffer (100 mmol/L pH 8.0).

The peptide concentrations were measured with a Quantitative Colorimetric Peptide Assay (Thermo Scientific) before TMT labeling to ensure that the amount of peptides in each channel for TMT labeling was equal. TMT6plex amino-reactive reagents (0.8 mg per vial, Thermo Scientific) were suspended in 41 μL of anhydrous acetonitrile and were suitable for labeling the 100 μg peptide solutions in each channel. Reactions were allowed to proceed at room temperature for 1 h and then quenched by the addition of 8 μL of 5% hydroxylamine for 15 min. The TMT-labeled samples were pooled at a 1:1:1:1:1:1 ratio. The mixture was vacuum centrifuged to near dryness and desalted on a MonoSpin™ C18 column (GL Science).

The desalted TMT-labeled peptides were subjected to offline prefractionation with a Thermo Fisher Pierce high pH reversed-phase separation kit (Cat. 84868) according to the manufacturer’s user guide. We separated the complex peptide mixture into 8-10 fractions.

All the fractions for each TMT experiment were analyzed by separation on a homemade 30 cm-long pulled-tip analytical column (75 μm ID x 360 μm OD, ReproSil-Pur C18-AQ 1.9 μm resin, Dr. Maisch GmbH). The column was then placed in-line with an Easy-nLC 1200 nano HPLC instrument (Thermo Scientific) for mass spectrometry analysis. The analytical column temperature was set at 55 °C during the experiments. The mobile phase and elution gradient used for peptide separation were as follows: 0.1% formic acid in water as buffer A and 0.1% formic acid in 80% acetonitrile as buffer B; 0-1 min, 5%–8% B; 1-104 min, 8%–35% B; 104-114 min, 35%–50% B, 114-115 min, 50%–100% B, 115-120 min, 100% B. The flow rate was set at 300 nL/min. Data-dependent tandem mass spectrometry (MS/MS) analysis was performed with an Orbitrap Eclipse Tribrid mass spectrometer (Thermo Scientific). Peptides eluted from the LC column were directly electrosprayed into the mass spectrometer with a distal 2.1-kV spray voltage. A 3 s cycle of one full-scan MS spectrum (m/z 400-1 600) was acquired, followed by acquisition in top speed mode (first mass fixed at a 100 m/z scan range) at a 38% normalized collision energy. The full scan resolution was set to 120 000 with an automated gain control (AGC) target of 4e5. The MS/MS scan resolution was set to 50,000 with an isolation window of 1.2 m/z and an AGC target of 1.25e5. The number of microscans was one for both the MS and MS/MS scans, and the maximum ion injection times for the MS and MS/MS scans were 50 and 100 ms, respectively. The dynamic exclusion settings used were as follows: charge exclusion, 1 and > 8; exclude isotopes, on; and exclusion duration, 60 s.

The acquired MS/MS data were analyzed against a UniProtKB human database (released on Sept. 30, 2018) by MaxQuant V1.6.10.43 using the default setting. The tolerance of the precursor mass and fragment mass were set to ± 20 ×10^−6^. The main search peptide tolerance was set at 4.5 ×10^−6^ according to the features of the instrument in the study. Carbamidomethylation of cysteine ( + 57.021 Da) and acetylation of the protein N-terminus were set as static modifications. The quantification search type selected was reporter ion ms2 using the TMT 10plex method. Oxidation of methionine ( + 15.995 Da) was set as a variable modification. Trypsin was defined as the cleavage enzyme, and the maximum number of missed cleavages was set at 2. All identified proteins had an FDR ≤ 1%, which was calculated at the peptide level. The unique peptides were selected for protein quantitation.

The interpretation of the proteomic results (protein Groups.txt) from MaxQuant was performed with the R4.0.0 platform. Briefly, P values were calculated by empirical Bayes moderated *t* tests and then adjusted by the Benjamini and Hochberg method using the limma package built for the R environment. Proteins were required to have a |log_2_ (fold change)| ≥ 0.26 and an adj.*P* value of 0.05 to be considered differentially expressed. Gene Ontology (GO) analysis was performed using the DAVID tool (https://david.ncifcrf.gov/).

### Immunofluorescence analysis with confocal microscopy

HNSCC cells were seeded into confocal dishes (NEST, China) with a φ of 20 mm and transfected with siRNA, plasmid or lentivirus when the cells reached 60%-70% confluence. At the set timepoint, the modified cells were fixed with 4% paraformaldehyde for 15 min, permeabilized in 0.1% Triton X-100 or saponin for 10 min, and then blocked with 1% BSA for 30 min. Primary antibodies against SEMA7A, FUT8, Calreticulin, GM130, TGN46, Giantin and HMGB1 were added and incubated overnight at 4 °C. After washing three times, the cells were then treated with Alexa Fluor 488- or Alexa Fluor 647-conjugated secondary antibodies (1:400, Jackson Lab) for 1 h at RT and subjected to nuclear staining with DAPI for 10 min; finally, images were captured with a CLSM (Leica SP8).

### Tissue microarray, IHC staining, and image analyses

The HNSCC tissue array with 70 samples was manufactured by Shanghai Outdo Biotech Co., Ltd. Immunohistochemical staining was performed following standard protocols. Specifically, pathological sections were deparaffinized and subjected to antigen retrieval using Tris-borate-EDTA. Subsequently, nonspecific protein interactions were blocked with 10% normal goat serum for 30 min. The sections were then incubated with primary antibodies (specific for SEMA7A and FUT8) overnight at 4 °C and then with horseradish peroxidase-conjugated secondary antibodies for 1 h at RT. The slides were washed with distilled water and then counterstained with hematoxylin. A pathologist performed semiquantitative analysis of IHC staining in a blinded manner by calculating the percentage of positive cells in at least five randomly selected fields. The staining results were used to categorize the samples into the high score (3x % of strongly stained cells) and low score (1x % of weakly stained cells) groups. Finally, the staining results were correlated with HNSCC prognosis.

### RIP-Seq

The RIP-Seq service was provided by Cloud-Seq Biotech (Shanghai, China). Briefly, the RNA immunoprecipitation assay was carried out with a GenSeq® RIP kit (GenSeq Inc., China) according to the manufacturer’s instructions. rRNAs were removed from the immunoprecipitated RNA and input RNA samples by using the NEBNext rRNA Depletion Kit (New England Biolabs, Inc., Massachusetts, USA). RNA libraries were constructed by using rRNA-depleted RNAs with the NEBNext® Ultra™ II Directional RNA Library Prep Kit (New England Biolabs, Inc., Massachusetts, USA) according to the manufacturer’s instructions. Libraries were controlled for quality and quantified using the BioAnalyzer 2100 system (Agilent Technologies, Inc., USA). Library sequencing was performed on an Illumina NovaSeq instrument with 150 bp paired-end reads. Briefly, paired-end reads were obtained with an Illumina NovaSeq 6000 sequencer, and Q30 was deemed to indicate sufficient quality control. Then, 3’ adaptor trimming and low-quality read removal were performed with Cutadapt software (v1.9.3). First, clean reads of all libraries were aligned to the reference genome (hg19) by Hisat2 software (v2.0.4). Genes were identified by MACS software with every pair. GO annotation and pathway enrichment analyses were performed with selected genes.

### Statistical analysis

Data in bar graphs are presented as the mean fold change values relative to untreated or control groups with standard deviations obtained from three independent experiments. Statistical analyses were performed using SPSS software (version 20, SPSS, Chicago, IL). Correlations between protein expression levels were analyzed using Spearman correlation analysis and the Mann‒Whitney test. One-way ANOVA with a post hoc Tukey test was used to determine the significance of differences between groups. A *P* value < 0.05 was considered statistically significant.

### Supplementary information

Supplementary tables and figures

Supplementary Data

## Data Availability

All data associated with this study are presented in the paper.
