# Author Correction: FUT8-mediated aberrant N-glycosylation of SEMA7A promotes head and neck squamous cell carcinoma progression

**DOI:** 10.1038/s41368-024-00307-x

**Published:** 2024-07-02

**Authors:** Zhonglong Liu, Xiaoyan Meng, Yuxin Zhang, Jingjing Sun, Xiao Tang, Zhiyuan Zhang, Liu Liu, Yue He

**Affiliations:** 1 Department of Oral Maxillofacial & Head and Neck Oncology, Shanghai Ninth People’s Hospital Affiliated to Shanghai Jiao Tong University School of Medicine, National Clinical Research Center for Oral Disease, Shanghai, China; 2 Department of Oral Surgery, Shanghai Ninth People’s Hospital, Shanghai Jiao Tong University School of Medicine; College of Stomatology, Shanghai Jiao Tong University; National Center for Stomatology; National Clinical Research Center for Oral Diseases, Shanghai, China; 3 Department of Oral Pathology, Shanghai Ninth People’s Hospital Affiliated to Shanghai Jiao Tong University School of Medicine, National Clinical Research Center for Oral Disease Shanghai, Shanghai, China

**Keywords:** Oral cancer, Cancer microenvironment

Correction to: *International Journal of Oral Science* 10.1038/s41368-024-00289-w, published online 28 March 2024

Following publication of the original article,^1^ the authors reported that some citations to supplementary figures were mistakenly written as citations to normal figures.

Below is a table of corrections which have been implemented in the original article:

**Table:** 

Location	Originally published text	Corrected text
Page 2	Fig. 1a	Supplementary Fig. 1a
Page 2	Fig. 1b, c	Supplementary Fig. 1b, c
Page 2	Fig. 2a	Supplementary Fig. 2a
Page 3	Fig. 2b	Supplementary Fig. 2b
Page 3	Figs. 1a, 2c	Fig. 1a, Supplementary Fig. 2c
Page 3	Fig. 2d	Supplementary Fig. 2d
Page 3	Figs. 1e, f, 2e	Fig. 1e, f, Supplementary Fig. 2e
Page 4	Fig. 3a, b	Fig. 3b, Supplementary Fig. 3a
Page 5	Fig. 3b	Supplementary Fig. 3b
Page 5	Fig. 3c	Supplementary Fig. 3c
Page 5	Fig. 3d	Supplementary Fig. 3d
Page 5	Fig. 4	Supplementary Fig. 4a
Page 5	Fig. 4c	Fig. 4c, Supplementary Fig. 4b
Page 6	Figs. 4d, e, 5	Fig. 4d, e, Supplementary Fig. 5
Page 6	Fig. 6	Supplementary Fig. 6
Page 7	Fig. 7	Supplementary Fig. 7
Page 7	Figs. 5e, 8	Fig. 5e, Supplementary Fig. 8
Page 8	Fig. 9a	Supplementary Fig. 9a
Page 8	Fig. 9b	Supplementary Fig. 9b
Page 8	Fig. 9c–e	Supplementary Fig. 9c–e
Page 8	Fig. 10a	Supplementary Fig. 10a
Page 8	Fig. 10b	Supplementary Fig. 10b
Page 11	Supplementary Fig. S13c	Supplementary Fig. 13c

The original article^1^ has been updated.
