# Supplementary material for: FUT8-mediated aberrant N-glycosylation of SEMA7A promotes head and neck squamous cell carcinoma progression
Source: Int J Oral Sci. 2024 Mar 28;16:26. doi: 10.1038/s41368-024-00289-w (PMC10978839; doi:10.1038/s41368-024-00289-w)
Supplement: Supplementary file 1 — Supplementary tables and figures [file 41368_2024_289_MOESM1_ESM.pdf]

Supplementary Table 1

GeneSymbol	pvalues	foldchange	Description
**ITGA3**	0.0092757	10.072801	Homo sapiens integrin, alpha 3 (antigen CD49C, alpha 3 subunit of VLA-3 receptor) (ITGA3), transcript variant a, mRNA [NM 002204]
**SEMA7A**	0.0062225	17.174493	Homo sapiens semaphorin 7A, GPI membrane anchor (John Milton Hagen blood group) (SEMA7A), transcript variant 1, mRNA [NM 003612]
**TMEM132A**	0.0083016	6.1006475	Homo sapiens transmembrane protein 132A (TMEM132A), transcript variant 1, mRNA [NM_017870]
**PCDH7**	0.0061459	6.6336476	Homo sapiens protocadherin 7 (PCDH7), transcript variant a, mRNA [NM_002589]
**GPR158**	0.0074394	12.830361	Homo sapiens G protein-coupled receptor 158 (GPR158), mRNA [NM_020752]
**CDH2**	0.0024728	9.7281727	Homo sapiens cadherin 2, type 1, N-cadherin (neuronal) (CDH2), mRNA [NM_001792]
**TMEM2**	0.0175124	8.8265024	Homo sapiens transmembrane protein 2 (TMEM2), transcript variant 1, mRNA [NM_013390]
**CD276**	0.0020694	4.3360986	Homo sapiens CD276 molecule (CD276), transcript variant 1, mRNA [NM_001024736]

Supplementary Table 2

	Uniprot	Majority.protein.IDs	WT-Reporter. intensity.count	5NQ-Reporter. intensity.count	Peptide. counts..unique	logFC	P.Value
1	P19338	NUCL_HUMAN	130	130	34;1	0.763879	8.29E-09
2	Q9BWF3	RBM4_HUMAN	25	25	13;11	0.6122728	3.85E-06
3	P52597	HNRPF_HUMAN	66	66	15	0.564242	2.07E-05
4	Q9UHX1	PUF60_HUMAN	41	41	21	0.5586333	2.50E-05
5	Q9UH03	SEPT3_HUMAN	2	2	1	0.5432554	4.15E-05
6	Q7L4I2	RSRC2_HUMAN	1	1	1	0.5234438	7.84E-05
7	Q70Z53	F10C1_HUMAN	2	2	2	0.5177853	9.36E-05
8	O95218	ZRAB2_HUMAN	23	23	13	0.5105352	0.0001172

Supplementary Table 3. Primer list.

Gene name	FORWARD	REVERSE
SEMA7A	CTGCCACGGTTGCCTCATGTC	TGGCTCGGCTGGATTAATGGATTG
ITGA3	GCAGCAGGCTCAGGCACATAC	CACTCGGCGTTGTAAGGTCCAC
B7-H3	GCACGGCTCTGTCACCATCAC	AGCTCCTGCATTCTCCTCCTCAC
TMEM2	CTACATCAGTTCCGTGCAGTC	TAAATTGTCCTGGGTTTACGGA
TMEM132A	GGTCTTCTTCCCAGCGAATAAG	TACAGAACCTGAGTTGCTTAGG
CDH2	AGGCGTCTGTAGAGGCTTCTGG	GAGGCTGTCCTTCATGCACATCC
PCDH7	TGGATATGTTCCTGCTGCTTTA	GTACTGGCTCTAGAACATCTCC
GPR158	CCATCACCTTCAGCACCGATTCG	AGGACAGGTCTTGGAGCAGGATG
Fut8	TGGTTCCTGGCGTTGGATTATGC	TGGACAGTTCTCGGCTAGAGTGATC
ST3GAL1	ACCATTTCCCACACCTACATC	CTTGCAGCCAGTTGTCAAAG
C2GNT1	AGCGGTATGAGGTCGTTAATG	ACATACCCCACATACTCCCTAC
C1GALT1	CTTCAATGCAGATTCTAGCCAAC	GTAGCTTTGACGTGTTTGGC
ST3GAL4	TCGTCATGGTGTGGTATTCC	CAGGAAGATGGGCTGATCC
ST6GAL1	TAGCAAGTGCAGCCTCACGA	AACCCAGCTGACAGGACAGG
MAGT5	TGTGAGGGAAAGATCAAGTGG	GCTCTCCAAGGTAAATGAGGAC
GALNT12	AGCCTGGTCAACTCTCCTTCG	GGGCAGTCCCGAAAGCTCAT
B3GAT2	GCCTTTTGCCATCGACATG	AGTCAGATTCTTGCATCCCTG
GCNT2	GCCTTGTGAAGAGATCATCC	CAGAATAGCCTGAAGCACTG
MGAT3	GAGTCCAACTTCACGGCTTAT	AGTGGTCCAGGAAGACATAGA
B3GNT2	GGCCTCTACCCACCCTATGC	AGGCCGAGTTTCTGAAGGCA
GCNT3	CCTGGCTCTGTTCCCAACCA	CAGATAGCCCGCTGGTGGAT
B3GAT1	CACCATCACCCTCCTTTCTATTC	GAACAACAGGTCTGGGATTTCT
B3GNT3	GCAACGCCTGTCCTCCTTTG	GATTGCCGCAGGTGAGGTTG
GALNT16	TGTGGATGTGTGGTGGCAGT	CACACTTCTGCAGTGCGCTT
Stx18	CCGCCACTGGGTCCAAGGAG	CAGAAGCACAGGCACGAGAGAAG
Fibronectin1	AGAGGCATAAGGTTCGGGAAGAGG	CGAGTCATCCGTAGGTTGGTTCAAG
Snail1	CCGAGGAATAGACATCATTCGA	CAGATGAGGCAGAATCTGGTAT
Vimentin	CCTTCGTGAATACCAAGACCTGCTC	AATCCTGCTCTCCTCGCCTTCC
N-cadherin	GGACAGTTCCTGAGGGATCAAAGC	CTTGGAGCCTGAGACACGATTCTG
Occludin	AACTTCGCCTGTGGATGACTTCAG	TTTGACCTTCCTGCTCTTCCCTTTG
E-cadherin	CTGATTCTGCTGCTCTTGCTGTTTC	GGTCCTCTTCTCCGCCTCCTTC
RBM4	GTTCTCGGCTCTGTGCGTTCAG	GCAGGAGAGGAAAGGAAAGGAAAGG
PD-L1	GCCGAAGTCATCTGGACAAG	AGTGTGCTGGTCACATTGAA
PD-L1 isoform A	ATGCTGCACTTCAGATCACAGA	TCACATCCATCATTCTCCCTTTT
PD-L1 isoform B	GGCATTTGCTGAACGCCCC	TGCTTGTCCAGATGACTTCGG
PD-L1 isoform C	TGATCAGCTATGGTGGTGCC	GTGCTAGGGGACAGTGTTAGA

Supplementary Table 4. Antibodies and reagents list.

Antibodies	Source	Identifier
SEMA7A	Santa Cruz Biotechnology	sc-374432
SEMA7A	Abcam	ab263900
GAPDH (1A6)	Bioworld Biotechnology	MB001H
β-actin	Cell Signaling Technology	#4970
Flag	Biorbyt Explore.Bioreagents.	orb22843
HA	Cell Signaling Technology	#3724
Myc	Cell Signaling Technology	#2276
Ubiquitin	Cell Signaling Technology	#20326
OS9	Abcam	ab109510
ERECL1	Abcam	ab129267
HRD1	Abcam	ab170901
Calreticulin	Abcam	ab92516
GM130	Abcam	ab52649
TGN46	Abcam	ab174280
Giantin	Abcam	ab37266
BIP	Cell Signaling Technology	#3177
Plexin C1	Santa Cruz Biotechnology	sc-390216
6x-His tag monoclonal antibody, Alexa Fluor 647	Invitrogen	# MA1-135-A647
Fut8	Santa Cruz Biotechnology	sc-271244
Stx18	Santa Cruz Biotechnology	sc-293067
α-Tubulin	Abcam	ab7291
Caspase 3	Cell Signaling Technology	#9662
Cleaved Caspase 3	Cell Signaling Technology	#9664
Lamin A/C	Cell Signaling Technology	#4777
Na/K ATPase	Cell Signaling Technology	#23565
PD-L1	Abcam	ab213524
InVivoPlus anti-mouse PD-L1	BioXcell	10F.9G2
PD-1	Abcam	ab227681
LAG3	Abcam	ab237720
TIM3	Abcam	ab246318
CTLA4	Abcam	ab237712
CD8	STEMCELLTM Technologies	60022AZ.1
CD3	Abcam	ab16669
HMGB1	Abcam	ab18256
ERK1/2	Abcam	ab184699
Phospho-ERK1/2	Cell Signaling Technology	#9101
STAT3	Cell Signaling Technology	#9139
Phospho-STAT3	Cell Signaling Technology	#9145
EGFR	Cell Signaling Technology	#4267
Phospho-EGFR	Cell Signaling Technology	#4407
RBM4	Cell Signaling Technology	#50703

rabbit IgG (H+L) DyLight^{TM} 800 4X PEG Conjugate	Cell Signaling Technology	#5151
mouse IgG (H+L) DyLight^{TM} 800 4X PEG Conjugate	Cell Signaling Technology	#5257

**Lectins**	**Source**	**Identifier**
Biotinylated Vicia Villosa Lectin (VVL)	Vector laboratories	B-1235
Biotinylated Sambucus Nigra Lectin (SNA)	Vector laboratories	B-1305
Biotinylated Phaseolus Vulgaris Leucoagglutinin (PHA-L)	Vector laboratories	B-1115
Biotinylated Concanavalin A (Con A)	Vector laboratories	B-1005
Biotinylated Lens Culinaris Agglutinin (LCA)	Vector laboratories	B-1045
DyLight 488 Streptavidin	Vector laboratories	SA-5488
DyLight 594 Streptavidin	Vector laboratories	SA-5594

**Chemicals, Peptides, and Recombinant Proteins**	**Source**	**Identifier**
Anti-Flag Dynabeads	Bimake.cn, China	B26102
Anti-HA Dynabeads	Bimake.cn, China	B26202
Anti-MYC Dynabeads	Bimake.cn, China	B26302
PNGase F	New England Biolabs	P0704S
O-Glycosidase	New England Biolabs	P0733S
Cycloheximide (CHX)	Selleck.cn	S7418
Tunicamycin (Tm)	Abcam	ab120296
Hydroxychloroquine Sulfate (HCQ)	Selleck.cn	S4430
MG132	Selleck.cn	S2619
Bortezomib (BTZ)	Selleck.cn	S1013
Brefeldin A (BFA)	Selleck.cn	S7046
Cell Counting Kit-8 (CCK8)	Dojindo	CK04
Annexin V-FITC Apoptosis Detection Kit	BD Falcon	556547
DAPI	Sigma Aldrich	10236276001
2F-Peracetyl-Fucose (2F-Fuc)	MCE MedChemExpress	HY-W096600
EGF	Peprotech	AF-100-15
TGF-β1	Peprotech	100-21
IGF	Peprotech	AF-100-11
HGF	Peprotech	100-39H
FGF	Peprotech	AF-100-17A
Pierce^{TM} Streptavidin Magnetic Beads	ThermoFisher Scientific	88816
Pierce^{TM} Protein A/G Magnetic Beads	ThermoFisher Scientific	88803
Gefitinib	Selleck.cn	S1025
Erlotinib	Selleck.cn	S1023
Doxorubicin	Selleck.cn	E2516
EasySep™ Human CD8+ T Cell Isolation Kit	STEMCELLTM Technologies	17953RF
ImmunoCult™-XF T Cell Expansion Medium	STEMCELLTM Technologies	10981

ImmunoCult Human CD3/CD28 T Cell Activator/CD3/CD28 T	STEMCELLTM Technologies	10971
Human Recombinant IL-2	STEMCELLTM Technologies	78036.1
Cell Fractionation Kit	Cell Signaling Technology	#11843

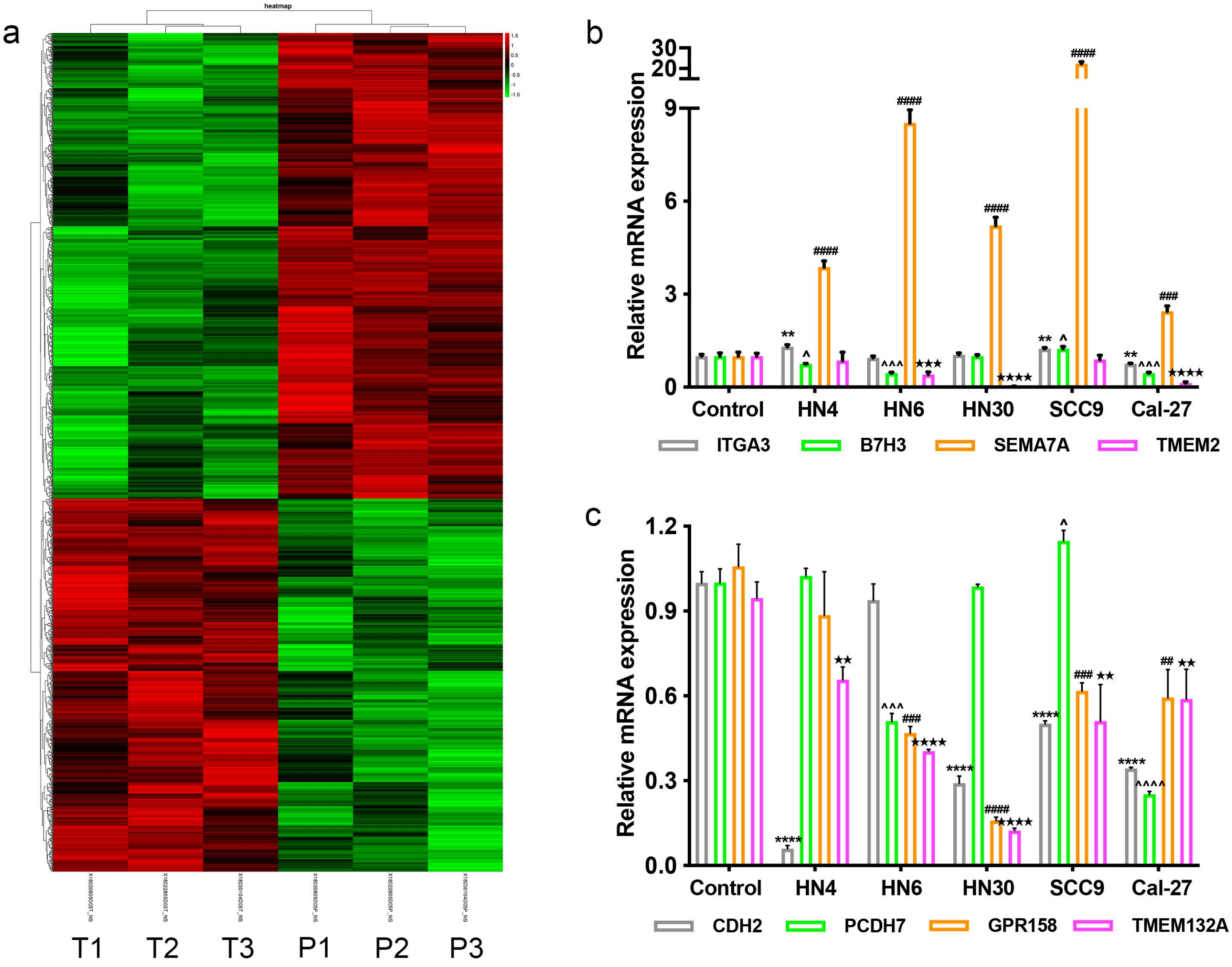
a
heatmap
T1
T2
T3
P1
P2
P3
b
Relative mRNA expression
Control
HN4
HN6
HN30
SCC9
Cal-27
ITGA3
B7H3
SEMA7A
TMEM2
c
Relative mRNA expression
Control
HN4
HN6
HN30
SCC9
Cal-27
CDH2
PCDH7
GPR158
TMEM132A

Supplementary Figure 1. **a** Heatmap presentation of different expression genes between tumors and adjacent normal tissues (n=3) on the basis of RNA-Sequence and subsequent bioinformatic analysis. **b-c** Verification of mRNA expression of 8 pivotal genes in five HNSCC cell lines (HN4, HN6, HN30, SCC9 and Cal-27) through real-time PCR analysis. (Data were shown as mean ± SEM, * $p \leqslant 0.05$,** $p \leqslant 0.01$, *** $p \leqslant 0.001$, **** $p \leqslant 0.0001$).

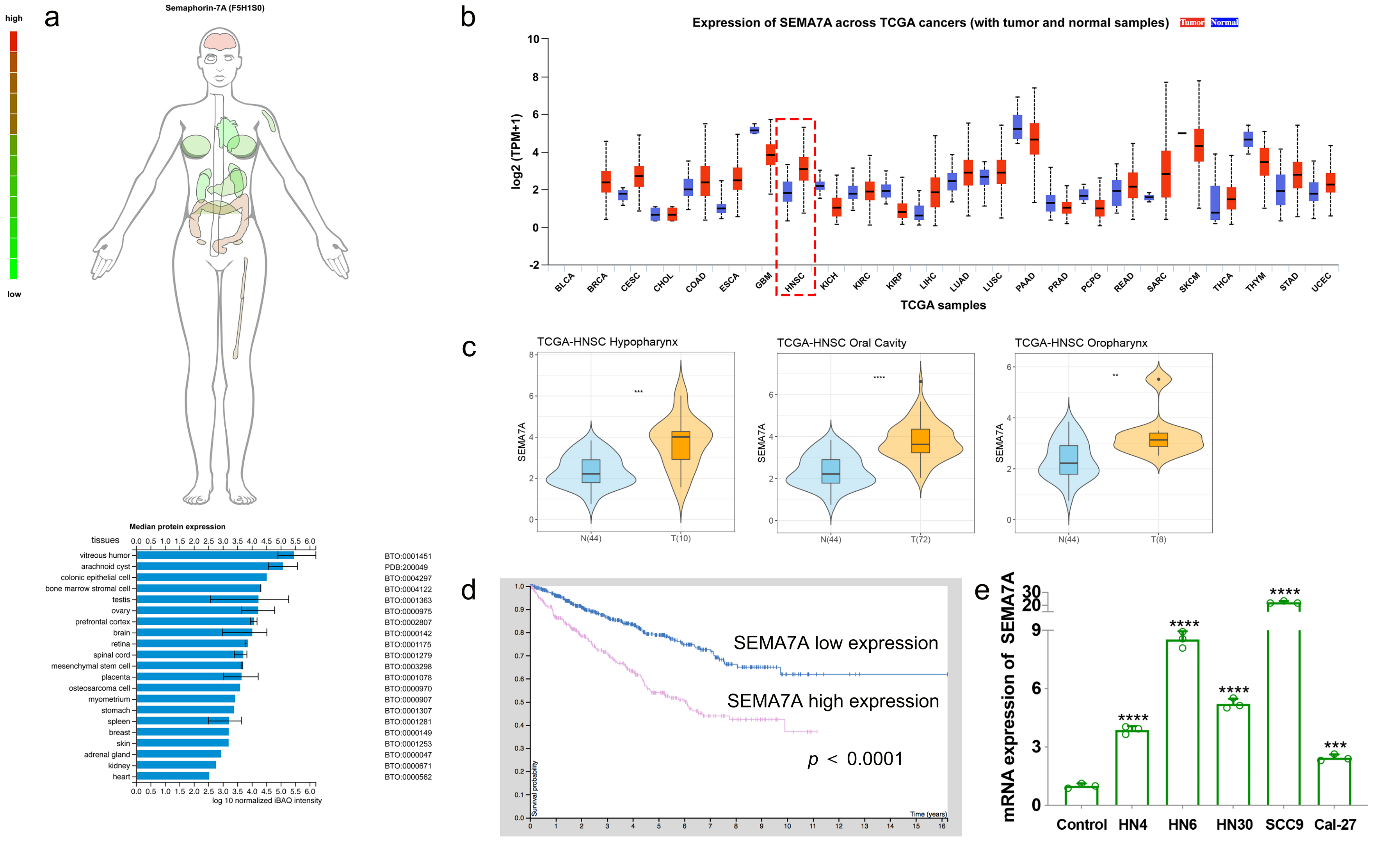
a
Semaphorin-7A (F5H1S0)
high
low
Median protein expression
tissues
0.0 0.5 1.0 1.5 2.0 2.5 3.0 3.5 4.0 4.5 5.0 5.5 6.0
vitreous humor
arachnoid cyst
colonic epithelial cell
bone marrow stromal cell
testis
ovary
prefrontal cortex
brain
retina
spinal cord
mesenchymal stem cell
placenta
osteosarcoma cell
myometrium
stomach
spleen
breast
skin
adrenal gland
kidney
heart
log 10 normalized iBAQ intensity
BTO:0001451
PDB:200049
BTO:0004297
BTO:0004122
BTO:0001363
BTO:0000975
BTO:0002807
BTO:0000142
BTO:0001175
BTO:0001279
BTO:0003298
BTO:0001078
BTO:0000970
BTO:0000907
BTO:0001307
BTO:0001281
BTO:0000149
BTO:0001253
BTO:0000047
BTO:0000671
BTO:0000562
b
Expression of SEMA7A across TCGA cancers (with tumor and normal samples)
Tumor
Normal
log2 (TPM+1)
BLCA
BRCA
CESC
CHOL
COAD
ESCA
GBM
HNSC
KICH
KIRC
KIRP
LIHC
LUAD
LUSC
PAAD
PRAD
PCPG
READ
SARC
SKCM
THCA
THYM
STAD
UCEC
TCGA samples
c
TCGA-HNSC Hypopharynx
TCGA-HNSC Oral Cavity
TCGA-HNSC Oropharynx
SEMA7A
***
****
**
N(44)
T(10)
T(72)
T(8)
d
SEMA7A low expression
SEMA7A high expression
p < 0.0001
Survival probability
Time (years)
e
mRNA expression of SEMA7A
****
***
Control
HN4
HN6
HN30
SCC9
Cal-27

Supplementary Figure 2. **a** Protein expression of SEMA7A in distinct organs and cell types across the entire body. **b** Differential expression of SEMA7A between tumors and adjacent tissues across pan-cancer in TCGA database. **c** Higher abundance of SEMA7A in tumors than adjacent tissues in TCGA-HNSCC across hypopharynx, oral cavity and oropharynx sites. **d** Kaplan–Meier curve of overall survival in TCGA-HNSCC patients stratified by the expression level of SEMA7A. **e** Overexpression of SEMA7A mRNA in different HNSCC cell lines testing by real-time PCR. (Data were shown as mean ± SEM, ** $p \leqslant 0.01$, *** $p \leqslant 0.001$, **** $p \leqslant 0.000$).

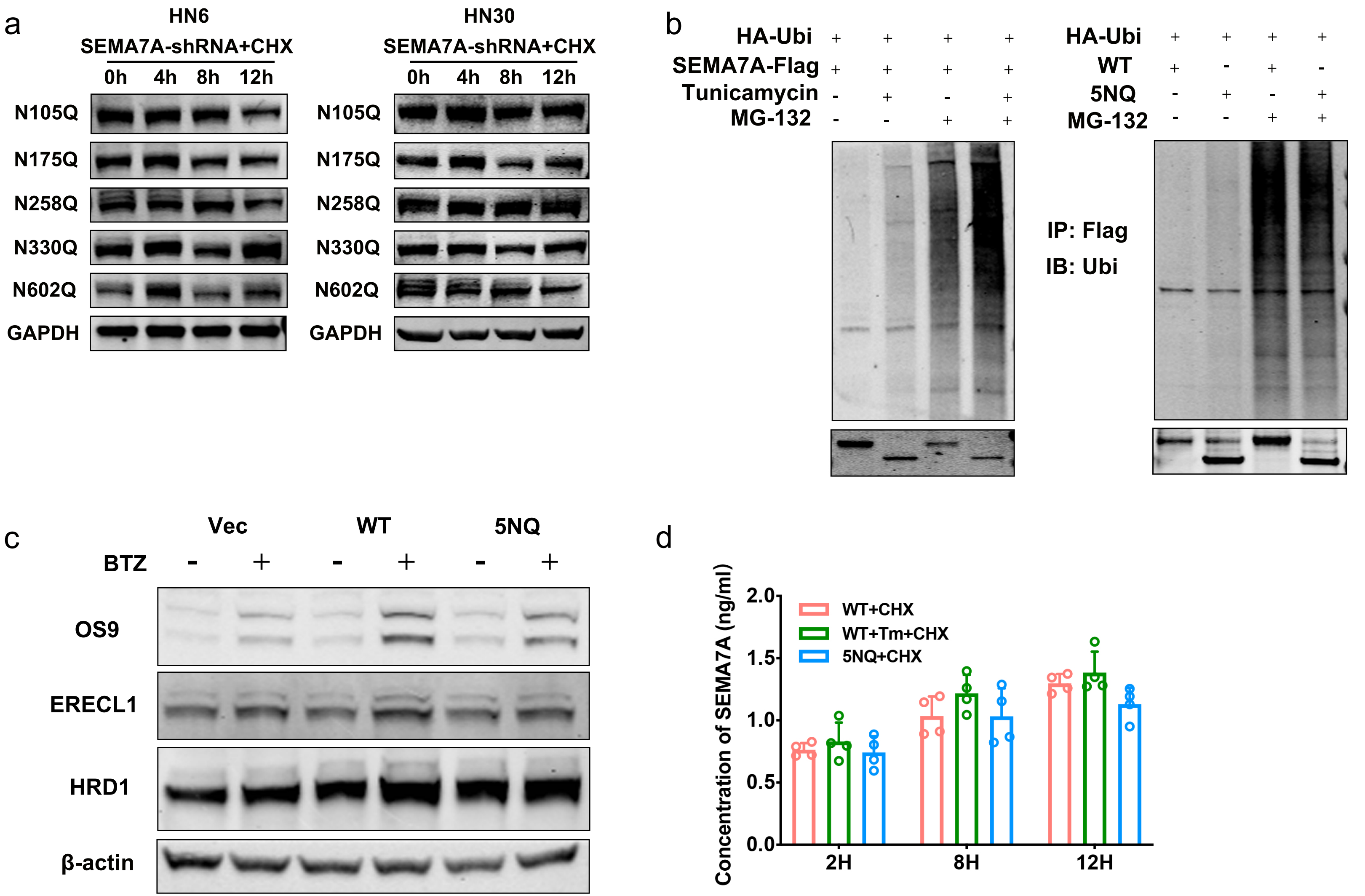

a
HN6
SEMA7A-shRNA+CHX
0h 4h 8h 12h
N105Q
N175Q
N258Q
N330Q
N602Q
GAPDH
HN30
SEMA7A-shRNA+CHX
0h 4h 8h 12h
b
HA-Ubi + + + +
SEMA7A-Flag + + + +
Tunicamycin - + - +
MG-132 - - + +
HA-Ubi + + + +
WT + - + -
5NQ - + - +
MG-132 - - + +
IP: Flag
IB: Ubi
c
Vec WT 5NQ
BTZ - + - + - +
OS9
ERECL1
HRD1
β-actin
d
Concentration of SEMA7A (ng/ml)
2.0
1.5
1.0
0.5
0.0
WT+CHX
WT+Tm+CHX
5NQ+CHX
2H
8H
12H

Supplementary Figure 3. **a** Lysates of SEMA7A-shRNA infected HN6 and HN30 cells expressing single N-glycosylation site mutant in the presence of CHX were measured at designed timepoints through western blot analysis. **b** HN6 cells were transiently transfected with distinct plasmids in the presence of tunicamycin and MG132 or not, and followed by flag-tag based immunoprecipitation detection of exogenous SEMA7A ubiquitination. **c** Comparison of the ERAD-related protein expression among HNSCC cells transfected with Vector, WT and 5NQ plasmids in the presence of BTZ or not. **d** Elisa measuring secretory SEMA7A from HNSCC cells expressing wild type or 5NQ mutant SEMA7A in the presence of CHX or tunicamycin at indicated time intervals.

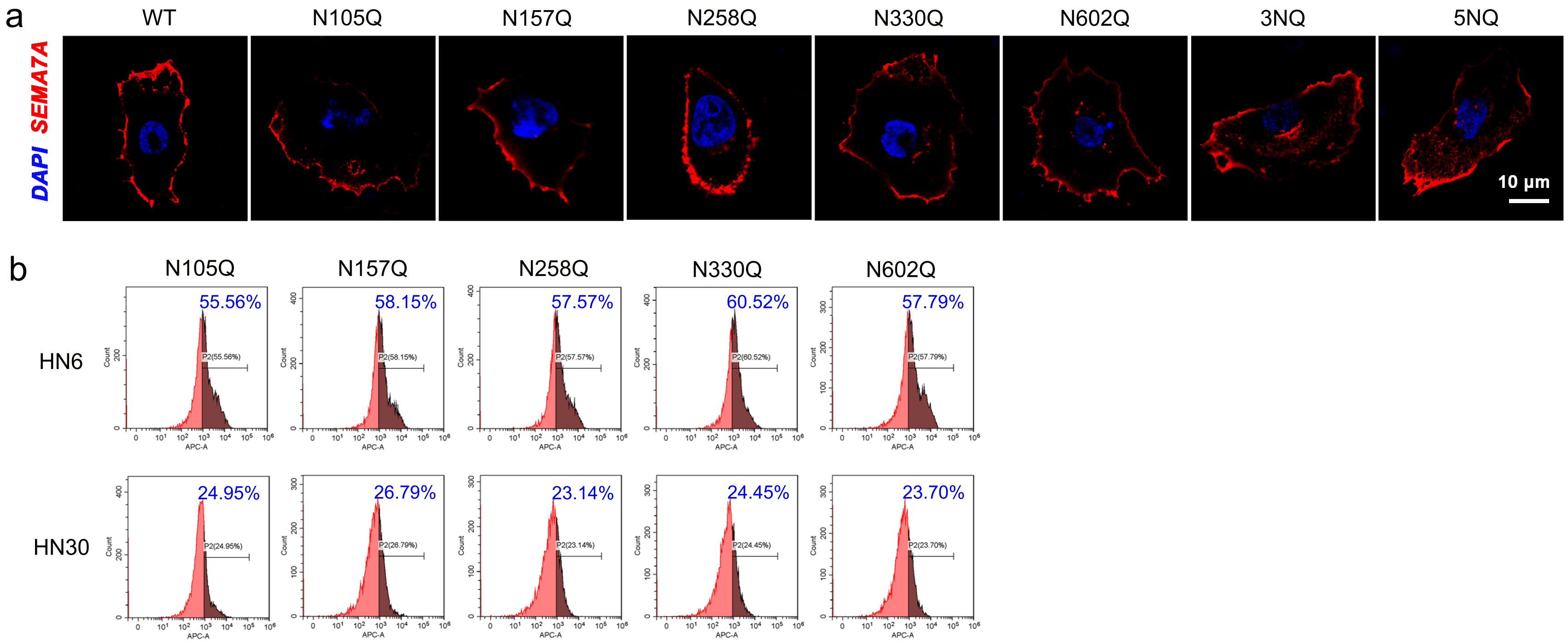
a
DAPI SEMA7A
WT
N105Q
N157Q
N258Q
N330Q
N602Q
3NQ
5NQ
10 μm
b
N105Q
N157Q
N258Q
N330Q
N602Q
HN6
55.56%
58.15%
57.57%
60.52%
57.79%
P2(55.56%)
P2(58.15%)
P2(57.57%)
P2(60.52%)
P2(57.79%)
HN30
24.95%
26.79%
23.14%
24.45%
23.70%
P2(24.95%)
P2(26.79%)
P2(23.14%)
P2(24.45%)
P2(23.70%)
Count
APC-A

Supplementary Figure 4. **a** Intracellular distribution of SEMA7A (Red) in HN6 cells transfected with wild type SEMA7A or distinct glycosylation site mutants through immunofluorescence observation. Nuclei were stained with DAPI. Scale bar: 10 μm. **b** Flow cytometry analysis the binding activity between SEMA7A with distinct glycosylated statues and Plexin C1 in HN6 and HN30 cells transfected with various mutation plasmids.

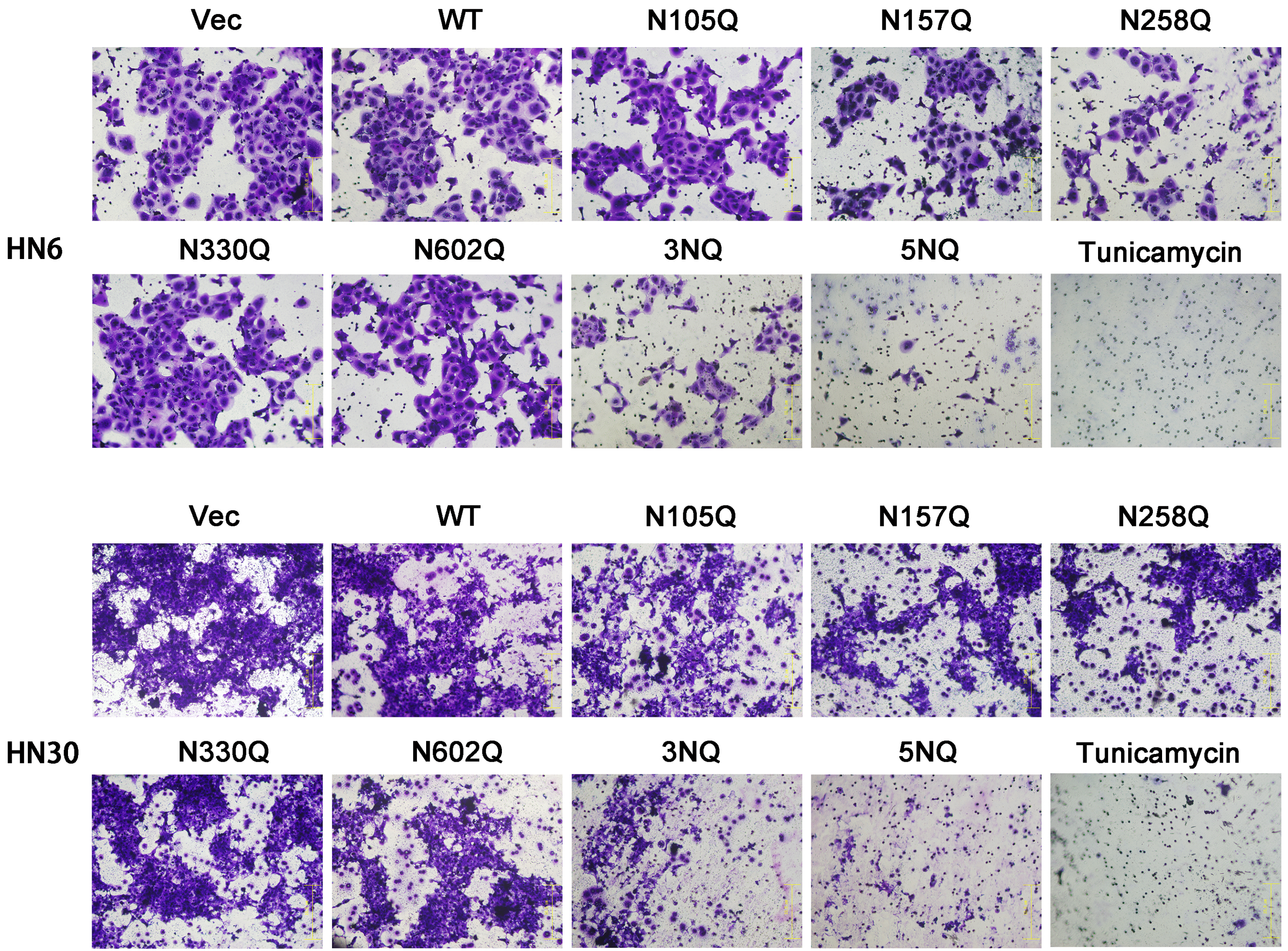
HN6
Vec
WT
N105Q
N157Q
N258Q
N330Q
N602Q
3NQ
5NQ
Tunicamycin
HN30
Vec
WT
N105Q
N157Q
N258Q
N330Q
N602Q
3NQ
5NQ
Tunicamycin

Supplementary Figure 5. Cell migration assay in HN6 and HN30 cells expressing wild type SEMA7A or different glycosylation site mutants or pre-treated with tunicamycin.

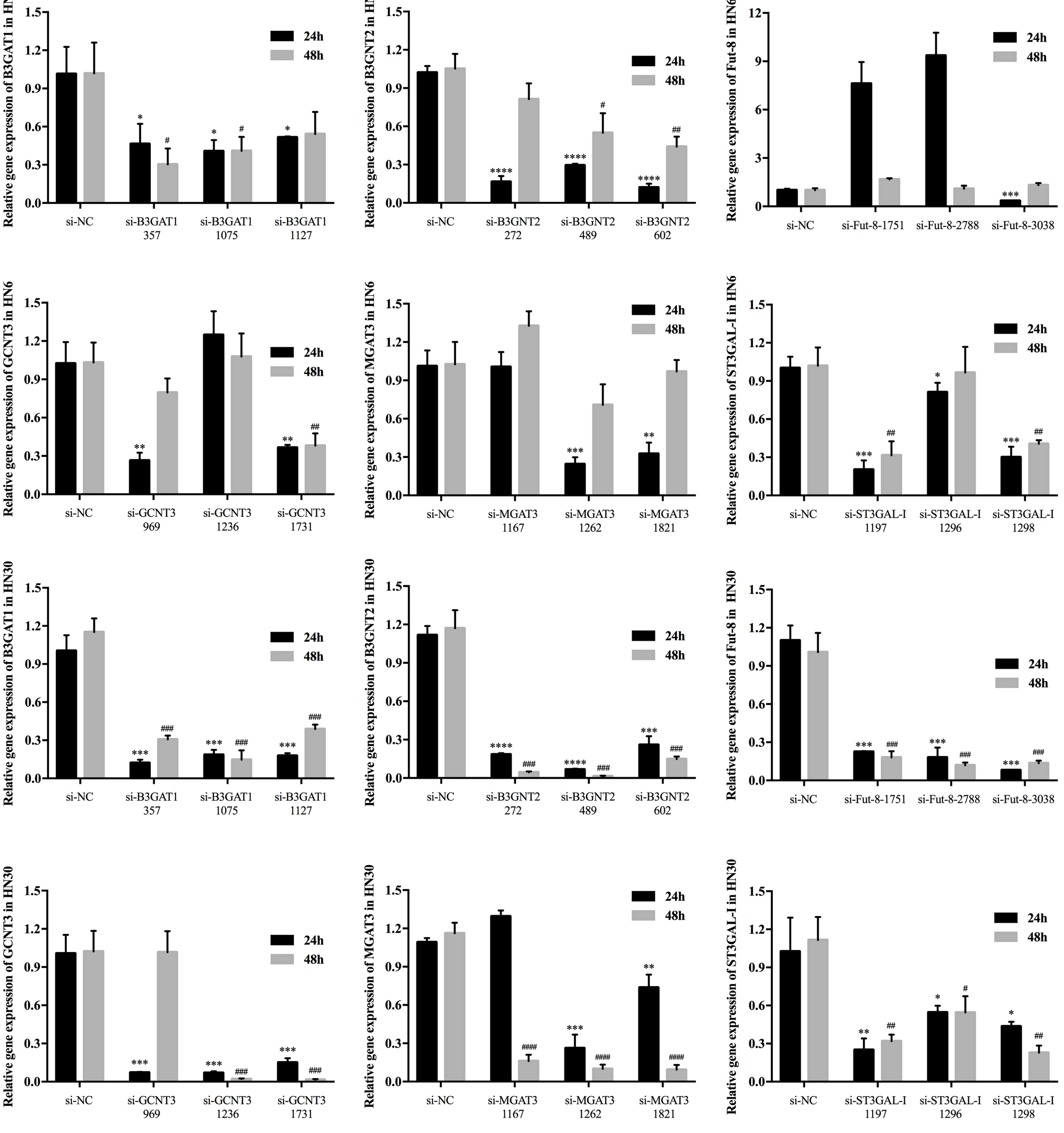
Relative gene expression of B3GAT1 in HN6
24h
48h
si-NC
si-B3GAT1 357
si-B3GAT1 1075
si-B3GAT1 1127
Relative gene expression of B3GNT2 in HN6
24h
48h
si-NC
si-B3GNT2 272
si-B3GNT2 489
si-B3GNT2 602
Relative gene expression of Fut-8 in HN6
24h
48h
si-NC
si-Fut-8-1751
si-Fut-8-2788
si-Fut-8-3038
Relative gene expression of GCNT3 in HN6
24h
48h
si-NC
si-GCNT3 969
si-GCNT3 1236
si-GCNT3 1731
Relative gene expression of MGAT3 in HN6
24h
48h
si-NC
si-MGAT3 1167
si-MGAT3 1262
si-MGAT3 1821
Relative gene expression of ST3GAL-I in HN6
24h
48h
si-NC
si-ST3GAL-I 1197
si-ST3GAL-I 1296
si-ST3GAL-I 1298
Relative gene expression of B3GAT1 in HN30
24h
48h
si-NC
si-B3GAT1 357
si-B3GAT1 1075
si-B3GAT1 1127
Relative gene expression of B3GNT2 in HN30
24h
48h
si-NC
si-B3GNT2 272
si-B3GNT2 489
si-B3GNT2 602
Relative gene expression of Fut-8 in HN30
24h
48h
si-NC
si-Fut-8-1751
si-Fut-8-2788
si-Fut-8-3038
Relative gene expression of GCNT3 in HN30
24h
48h
si-NC
si-GCNT3 969
si-GCNT3 1236
si-GCNT3 1731
Relative gene expression of MGAT3 in HN30
24h
48h
si-NC
si-MGAT3 1167
si-MGAT3 1262
si-MGAT3 1821
Relative gene expression of ST3GAL-I in HN30
24h
48h
si-NC
si-ST3GAL-I 1197
si-ST3GAL-I 1296
si-ST3GAL-I 1298

Supplementary Figure 6. Verification of knockdown efficiency of 6 N-glycosyltransferases siRNA (B3GAT1, B3GNT2, FUT8, GCNT3, MGAT3 and ST3GAL-I) in HN6 and HN30 cells through real-time PCR analysis. Data was shown as the mean ± SD, *P* value was calculated by Student's *t* test ($^{*}p < 0.05$, $^{**}p < 0.01$, $^{***}p < 0.001$, $^{****}p < 0.0001$; $^{\#}p < 0.05$, $^{\#\#}p < 0.01$, $^{\#\#\#}p < 0.001$, $^{\#\#\#\#}p < 0.0001$).

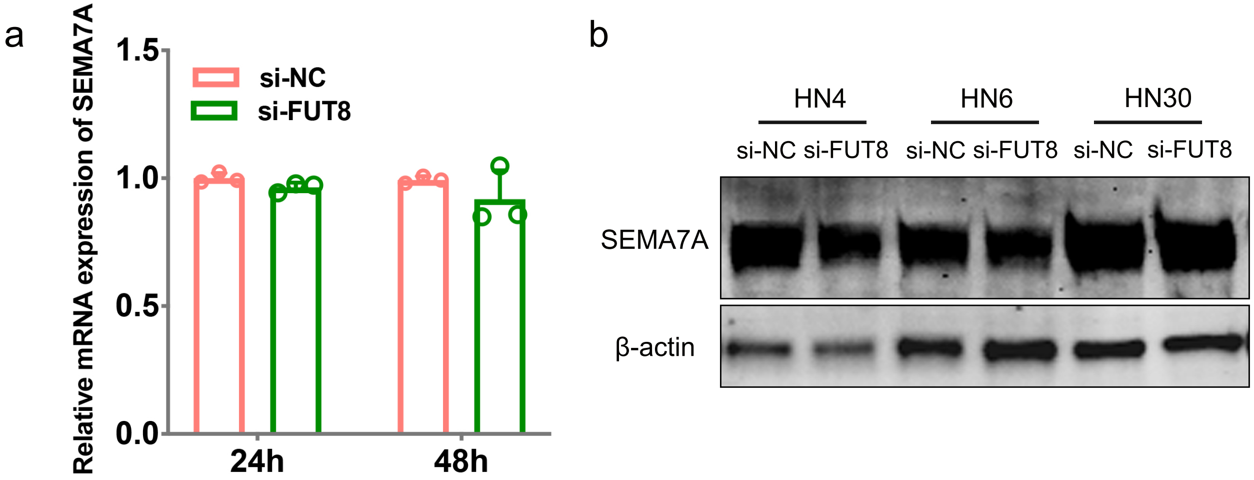
a
Relative mRNA expression of SEMA7A
1.5
1.0
0.5
0.0
si-NC
si-FUT8
24h
48h
b
HN4
HN6
HN30
si-NC si-FUT8 si-NC si-FUT8 si-NC si-FUT8
SEMA7A
β-actin

Supplementary Figure 7. **a** The influence of FUT8 knockdown on the mRNA expression of SEMA7A using real-time PCR analysis. **b** Downregulation of FUT8 through siRNA has no determined role on the protein expression of SEMA7A in different HNSCC cell lines.

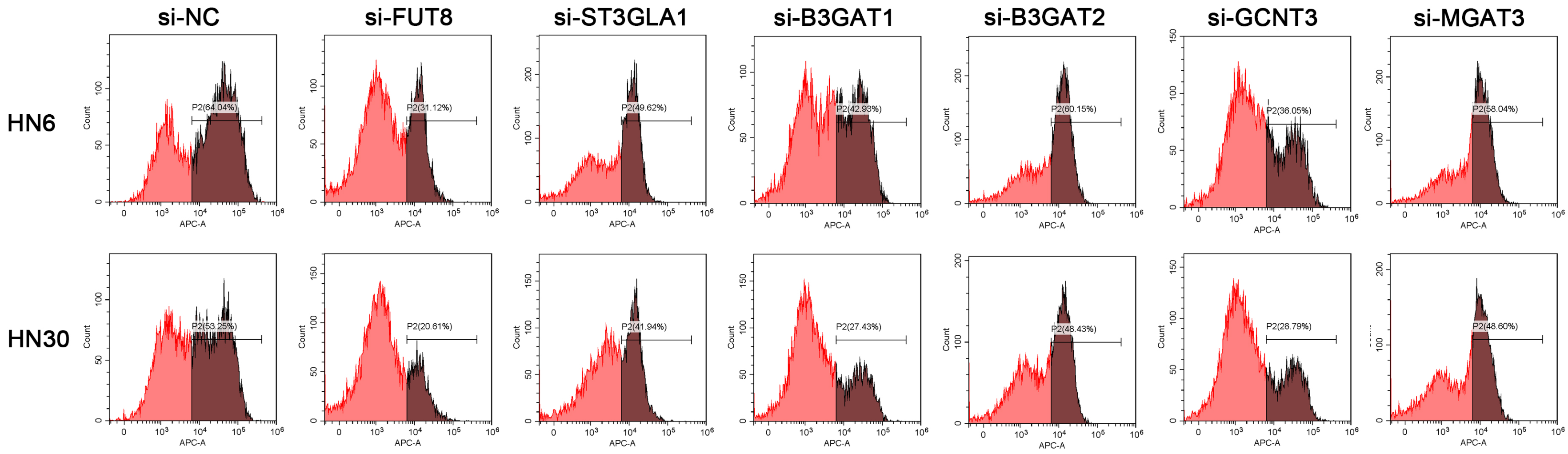
si-NC
si-FUT8
si-ST3GLA1
si-B3GAT1
si-B3GAT2
si-GCNT3
si-MGAT3
HN6
HN30
P2(64.04%)
P2(31.12%)
P2(49.62%)
P2(42.93%)
P2(60.15%)
P2(36.05%)
P2(58.04%)
P2(53.25%)
P2(20.61%)
P2(41.94%)
P2(27.43%)
P2(48.43%)
P2(28.79%)
P2(48.60%)
Count
APC-A

Supplementary Figure 8. Flow cytometry measuring the formation of SEMA7A/Plexin C1 complex in HN6 and HN30 cells transfected with si-NC or 6 N-glycosyltransferases siRNAs.

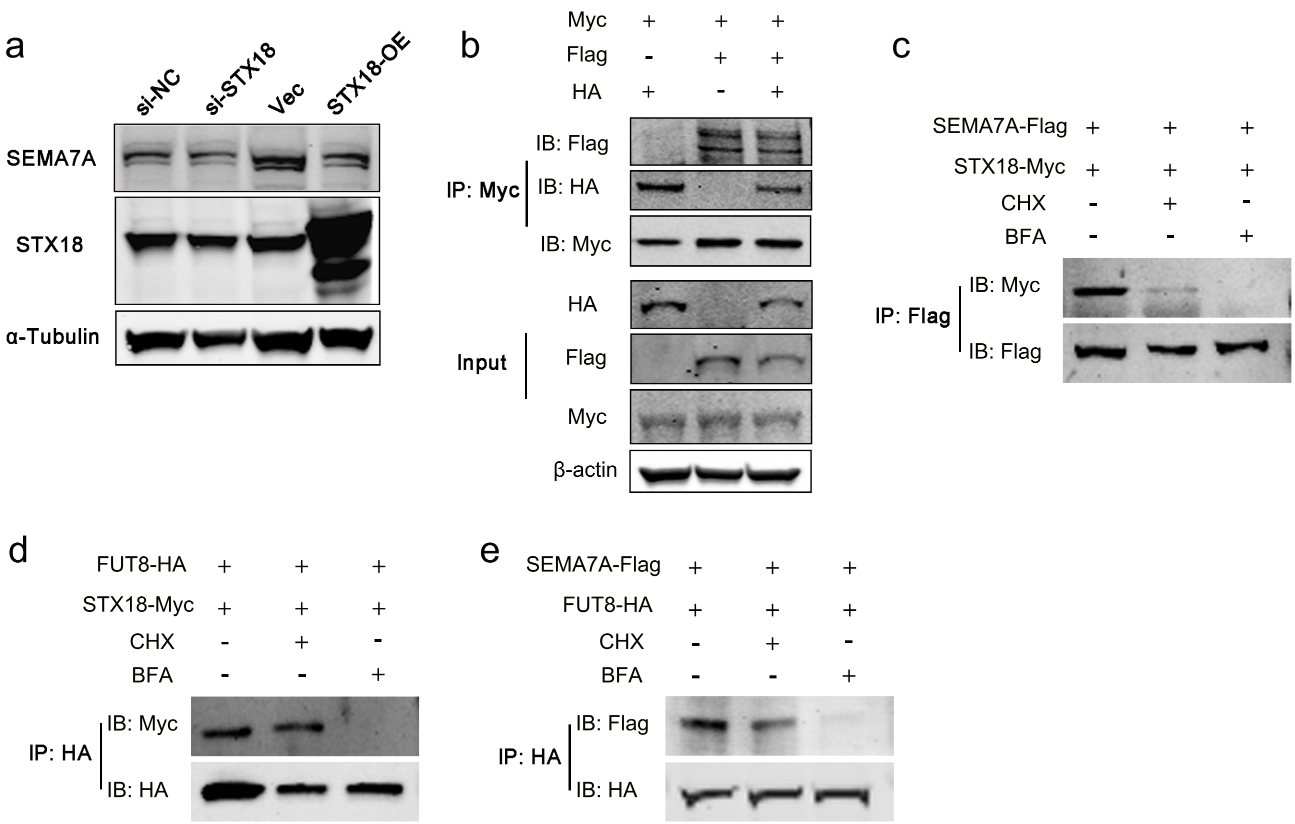
a
si-NC
si-STX18
Vec
STX18-OE
SEMA7A
STX18
α-Tubulin
b
Myc + + +
Flag - + +
HA + - +
IB: Flag
IP: Myc
IB: HA
IB: Myc
HA
Input
Flag
Myc
β-actin
c
SEMA7A-Flag + + +
STX18-Myc + + +
CHX - + -
BFA - - +
IP: Flag
IB: Myc
IB: Flag
d
FUT8-HA + + +
STX18-Myc + + +
CHX - + -
BFA - - +
IP: HA
IB: Myc
IB: HA
e
SEMA7A-Flag + + +
FUT8-HA + + +
CHX - + -
BFA - - +
IP: HA
IB: Flag
IB: HA

Supplementary Figure 9. **a** STX18 intervention has no influence on the protein expression of SEMA7A. **b** HN6 cells were transiently transfected with Flag-tagged SEMA7A-WT, HA-tagged FUT8 and Myc-tagged Stx8, followed by immunoprecipitation with anti-Myc beads and immunoblot analysis with anti-Flag, HA and Myc. **c-e** IP analysis of the interaction of exogenous SEMA7A-WT-Flag, FUT8-WT-HA with STX18-Myc in the presence or absence of CHX and BFA.

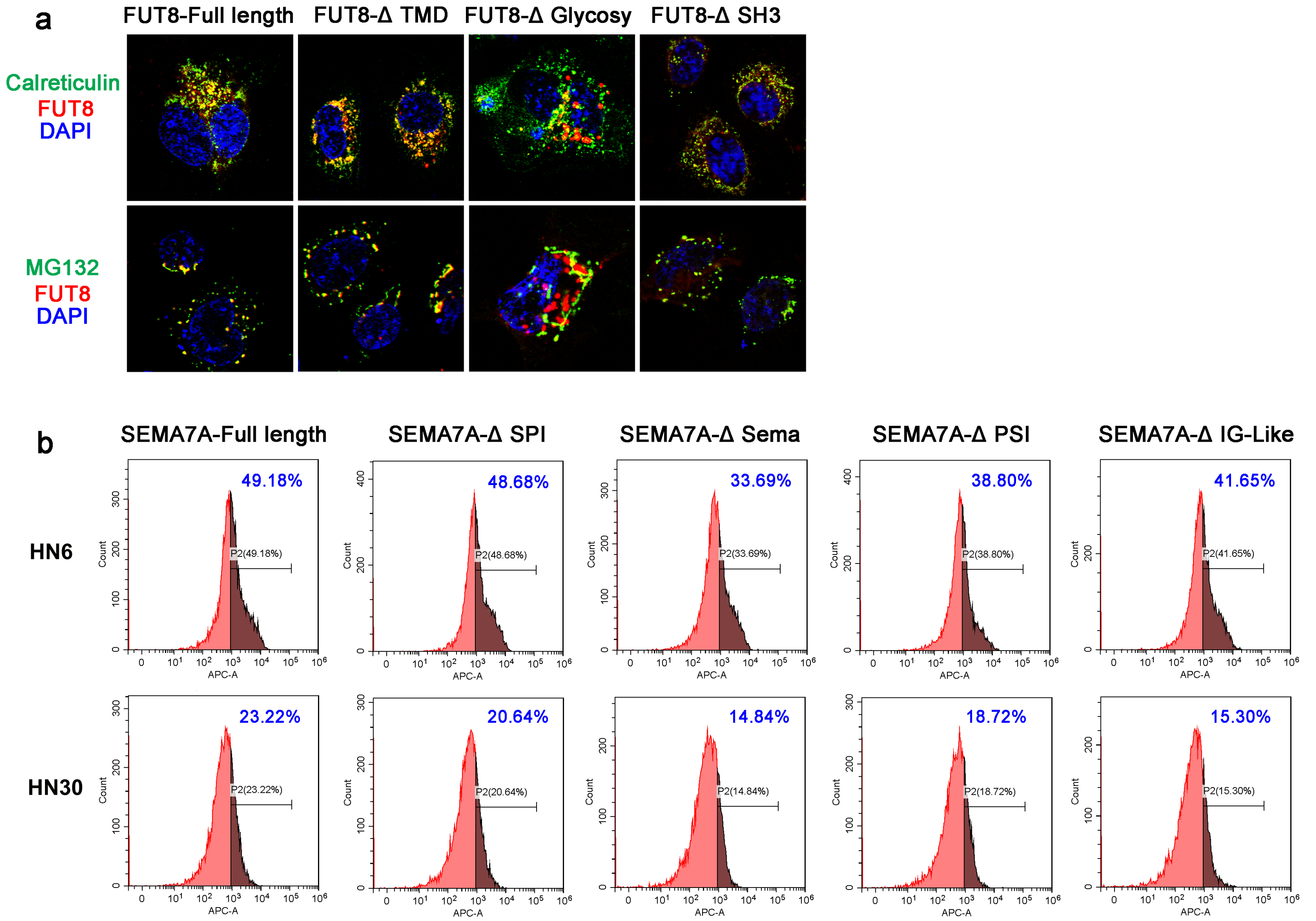

a
FUT8-Full length
FUT8-Δ TMD
FUT8-Δ Glycosy
FUT8-Δ SH3
Calreticulin
FUT8
DAPI
MG132
FUT8
DAPI
b
SEMA7A-Full length
SEMA7A-Δ SPI
SEMA7A-Δ Sema
SEMA7A-Δ PSI
SEMA7A-Δ IG-Like
HN6
HN30
49.18%
48.68%
33.69%
38.80%
41.65%
23.22%
20.64%
14.84%
18.72%
15.30%
P2(49.18%)
P2(48.68%)
P2(33.69%)
P2(38.80%)
P2(41.65%)
P2(23.22%)
P2(20.64%)
P2(14.84%)
P2(18.72%)
P2(15.30%)
Count
APC-A

Supplementary Figure 10. **a** Immunofluorescence observation of FUT8 distribution (red) in HNSCC cells expressing full length FUT8 or truncated FUT8 fragments through co-staining with ER (calreticulin, green) and Golgi (MG132, green) markers. **b** Flow cytometry measuring the binding affinity of SEMA7A with Plexin C1 in HN6 and HN30 cells expressing full length SEMA7A or truncated SEMA7A fragments.

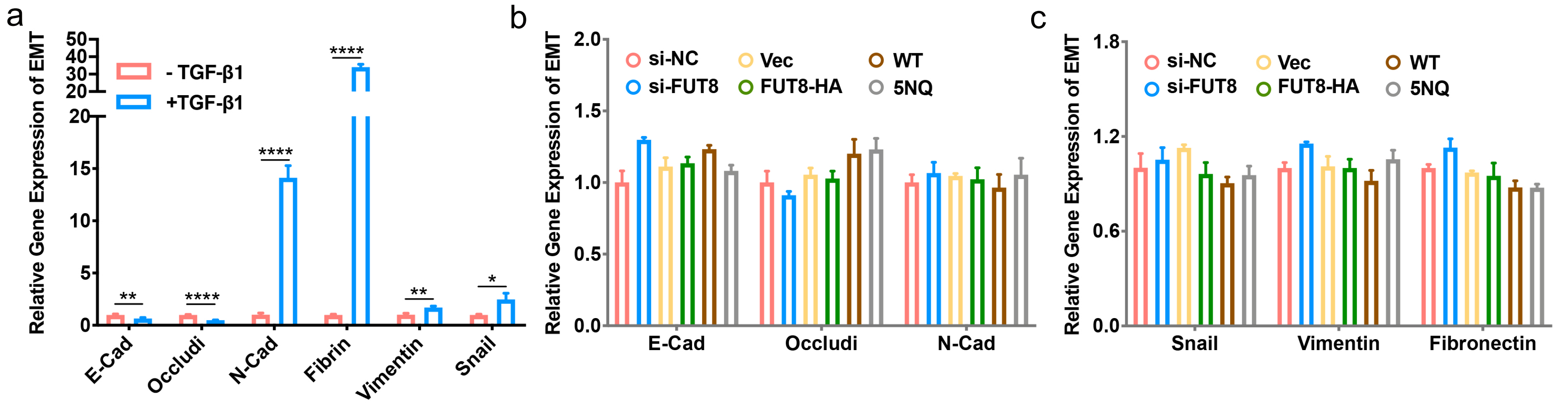

a
b
c
Relative Gene Expression of EMT
- TGF-β1
+TGF-β1
**
****
****
****
**
*
E-Cad
Occludi
N-Cad
Fibrin
Vimentin
Snail
si-NC
si-FUT8
Vec
FUT8-HA
WT
5NQ
E-Cad
Occludi
N-Cad
Snail
Vimentin
Fibronectin

Supplementary Figure 11. **a** mRNA expression of EMT related gene markers in HNSCC cells during TGF-β1 induction through real-time PCR analysis. **b-c** The influence of FUT8 or SEMA7A intervention on the TGF-β1 induced EMT process. (Data were shown as mean ± SEM, * $p \leqslant 0.05$,** $p \leqslant 0.01$, **** $p \leqslant 0.0001$).

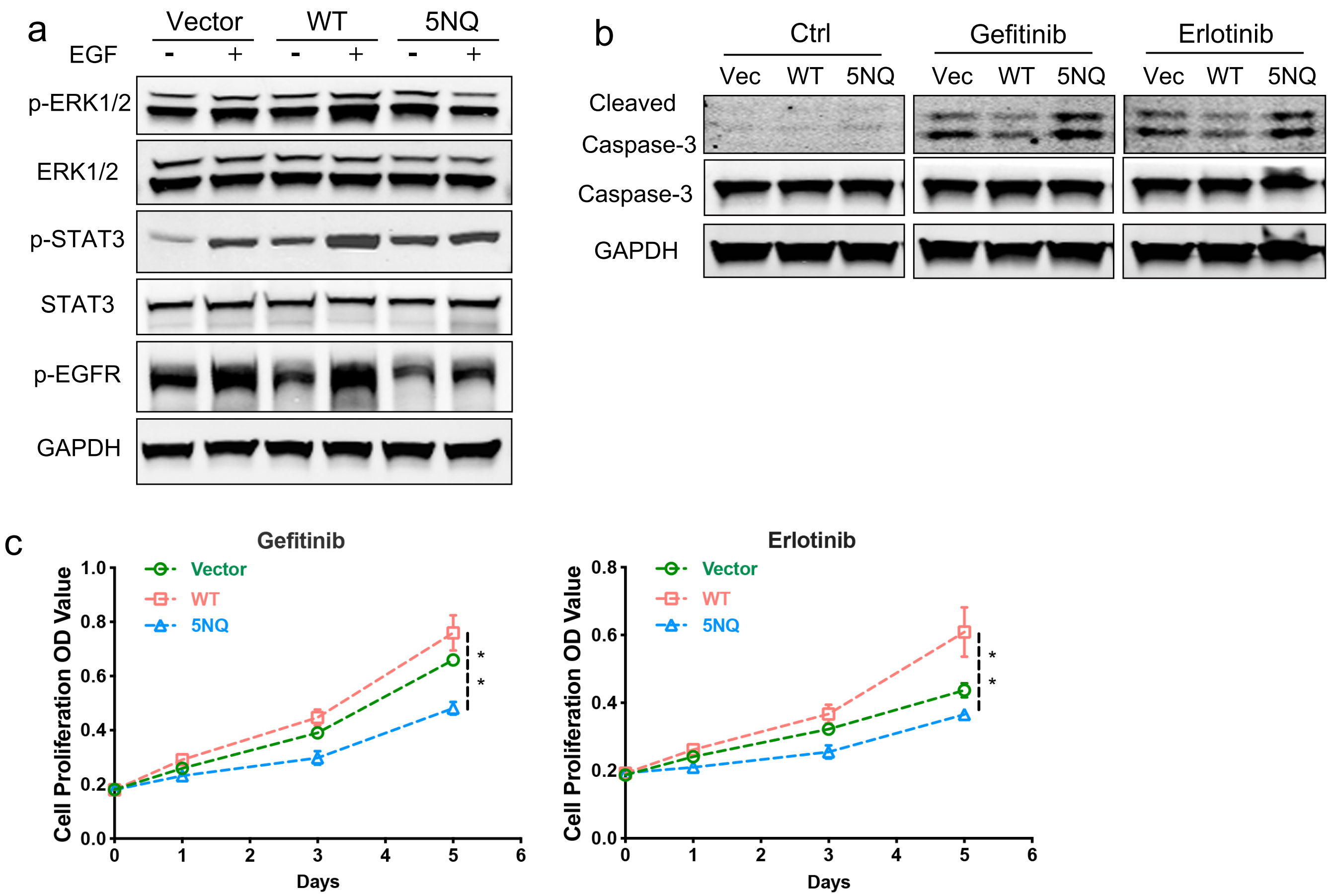

a
Vector
WT
5NQ
EGF
- + - + - +
p-ERK1/2
ERK1/2
p-STAT3
STAT3
p-EGFR
GAPDH
b
Ctrl
Gefitinib
Erlotinib
Vec WT 5NQ
Cleaved Caspase-3
Caspase-3
GAPDH
c
Gefitinib
Erlotinib
Vector
WT
5NQ
Cell Proliferation OD Value
Days
*
*

Supplementary Figure 12. **Deglycosylation of SEMA7A promoted EGFR targeted therapy in HNSCC cells. a** EGFR signaling was inhibited in HN6 cells expressing SEMA7A-5NQ mutant compared to that transfected with SEMA7A-WT in the presence of EGF. **b** Cellular apoptosis related markers detection in HN6 cells expressing wild type SEMA7A or 5NQ mutant in the presence of Gefitinib and Erlotinib or not. **c** *In vitro* cell proliferation measuring in SEMA7A-WT or 5NQ transfecting HN6 cells cotreatment with Gefitinib and Erlotinib at designed timepoints. Data was shown as the mean ± SD, p value was calculated by Student's t test ($^{**}p < 0.01$).

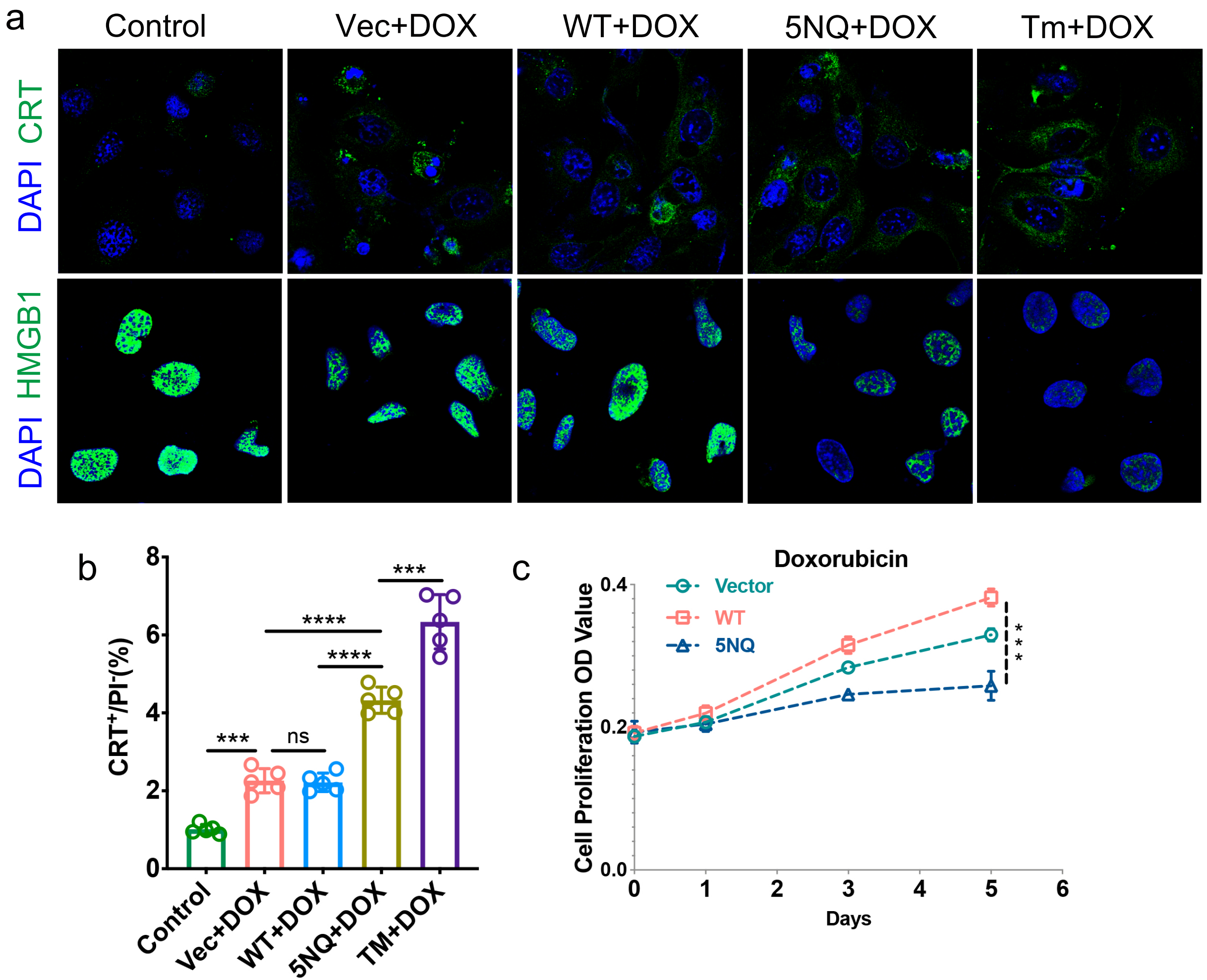
a
Control
Vec+DOX
WT+DOX
5NQ+DOX
Tm+DOX
DAPI CRT
DAPI HMGB1
b
CRT+/PI-(%)
***
ns
****
****
***
Control
Vec+DOX
WT+DOX
5NQ+DOX
TM+DOX
c
Doxorubicin
Vector
WT
5NQ
***
Cell Proliferation OD Value
Days

Supplementary Figure 13 The influence of SEMA7A glycosylation on immunogenic cell death (ICD). a Representative confocal staining of calreticulin and HMGB1 in HN6 cells transfected with SEMA7A-WT and 5NQ followed by Dox treatment. **b** Statistical analysis of calreticulin positive cells in HN6 cells from various groups. **c** Cell viability analysis of HNSCC cells transfected with SEMA7A-WT and 5NQ followed by Doxorubicin treatment. (Data were shown as mean ± SEM, ***$p \leq 0.001$, ****$p \leq 0.0001$).

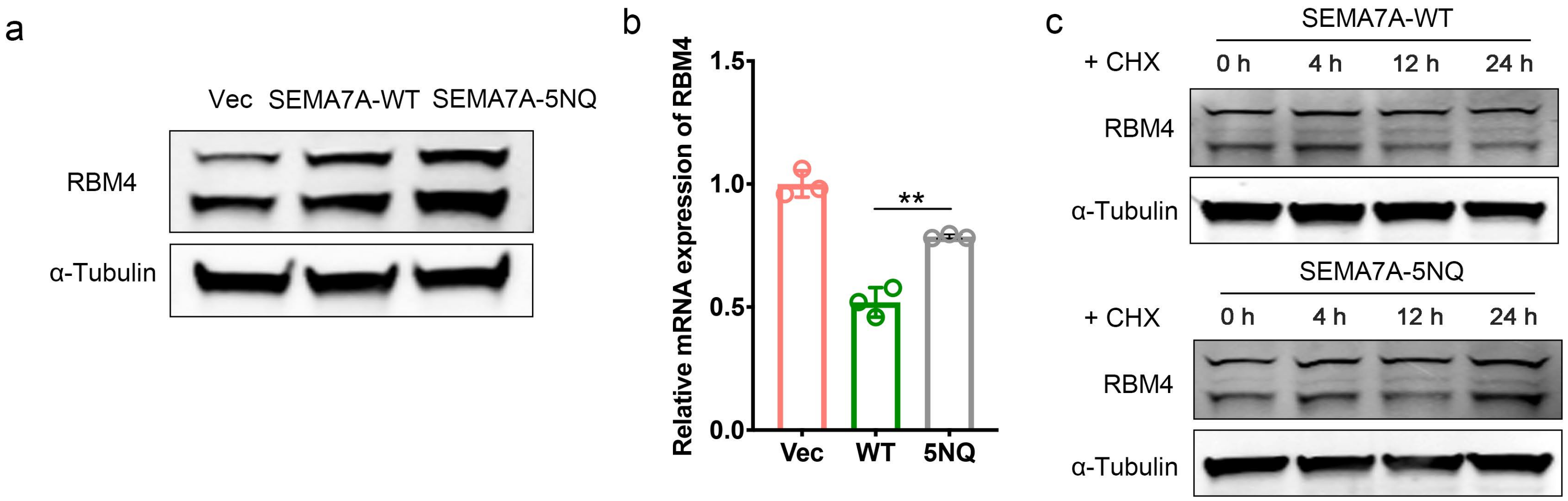
a
Vec
SEMA7A-WT
SEMA7A-5NQ
RBM4
α-Tubulin
b
Relative mRNA expression of RBM4
1.5
1.0
0.5
0.0
**
Vec
WT
5NQ
c
SEMA7A-WT
+ CHX
0 h
4 h
12 h
24 h
RBM4
α-Tubulin
SEMA7A-5NQ
+ CHX
0 h
4 h
12 h
24 h
RBM4
α-Tubulin

Supplementary Figure 14. **a** Relative protein expression of RBM4 in HN30 cells expressing wild type SEMA7A or 5NQ mutant through western blot. **b** Relative mRNA expression of RBM4 in HN6 cells expressing wild type SEMA7A or 5NQ mutant through real-time PCR. **c** The protein degradation of RBM4 in SEMA7A-WT or 5NQ transfecting HN6 cells in the presence of CHX at indicated time intervals. (Data were shown as mean ± SEM, ** $p \leqslant 0.01$).

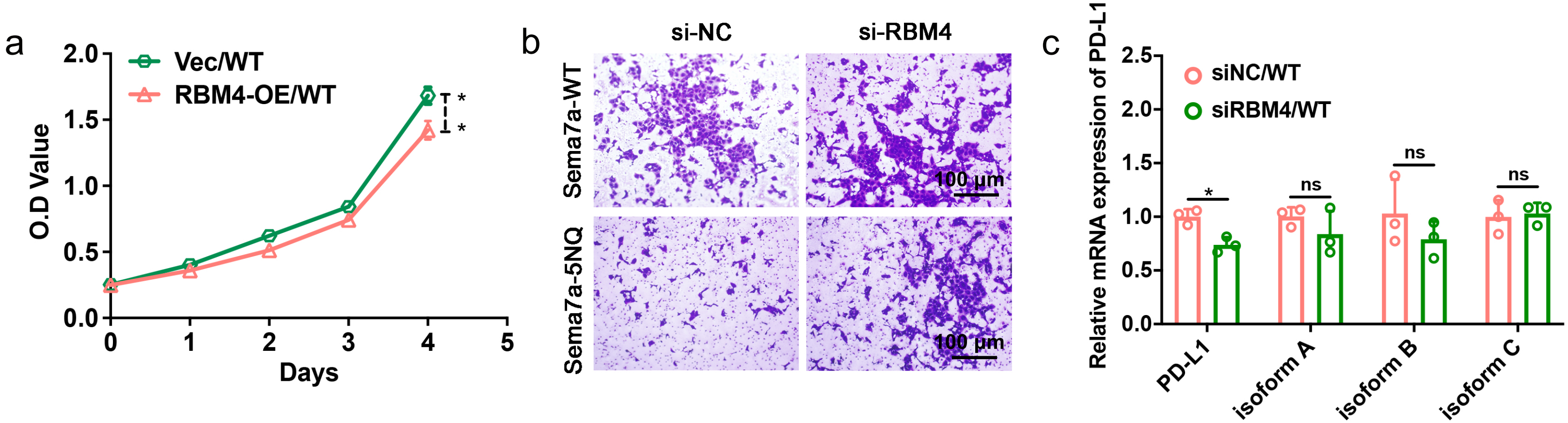
a
O.D Value
Days
Vec/WT
RBM4-OE/WT
b
si-NC
si-RBM4
Sema7a-WT
Sema7a-5NQ
100 μm
c
Relative mRNA expression of PD-L1
siNC/WT
siRBM4/WT
PD-L1
isoform A
isoform B
isoform C
ns

Supplementary Figure 15. **a** Cell viability observation of HN6 cells overexpressed with SEMA7A-WT followed by Vector and RBM4-OE plasmids. **b** Transwell migration assay in HNSCC cells sequentially transfected with SEMA7A-WT/5NQ and si-NC/si-RBM4. **c** Transcriptive level of PD-L1 isoforms in HN6 cells sequentially transfected with SEMA7A-WT and si-NC/si-RBM4. (Data were shown as mean ± SEM, * $p \leqslant 0.05$,** $p \leqslant 0.01$).
